# Reliability of animal counts and implications for the interpretation of trends

**DOI:** 10.1002/ece3.7191

**Published:** 2021-01-27

**Authors:** David Vallecillo, Michel Gauthier‐Clerc, Matthieu Guillemain, Marion Vittecoq, Philippe Vandewalle, Benjamin Roche, Jocelyn Champagnon

**Affiliations:** ^1^ Tour du Valat Research institute for the conservation of Mediterranean wetlands Arles France; ^2^ OFB Unité Avifaune migratrice La Tour du Valat Arles France; ^3^ Chrono‐Environnement UMR CNRS Université de Franche‐Comté Besançon France; ^4^ SNPN‐RNN de Camargue Arles France; ^5^ IRD Sorbonne Université UMMISCO Bondy France; ^6^ MIVEGEC, IRD CNRS Université Montpellier Montpellier France; ^7^ Departamento de Etología Fauna Silvestre y Animales de Laboratorio Facultad de Medicina Veterinaria y Zootecnia Universidad Nacional Autónoma de México (UNAM) Ciudad de México México

**Keywords:** group size estimation error, population monitoring, sampling design, statistical power, time series

## Abstract

1. Population time series analysis is an integral part of conservation biology in the current context of global changes. To quantify changes in population size, wildlife counts only provide estimates because of various sources of error. When unaccounted for, such errors can obscure important ecological patterns and reduce confidence in the derived trend. In the case of highly gregarious species, which are common in the animal kingdom, the estimation of group size is an important potential bias, which is characterized by high variance among observers. In this context, it is crucial to quantify the impact of observer changes, inherent to population monitoring, on i) the minimum length of population time series required to detect significant trends and ii) the accuracy (bias and precision) of the trend estimate.
2. We acquired group size estimation error data by an experimental protocol where 24 experienced observers conducted counting simulation tests on group sizes. We used this empirical data to simulate observations over 25 years of a declining population distributed over 100 sites. Five scenarios of changes in observer identity over time and sites were tested for each of three simulated trends (true population size evolving according to deterministic models parameterized with declines of 1.1%, 3.9% or 7.4% per year that justify respectively a “declining,” “vulnerable” or “endangered” population under IUCN criteria).
3. We found that under realistic field conditions observers detected the accurate value of the population trend in only 1.3% of the cases. Our results also show that trend estimates are similar if many observers are spatially distributed among the different sites, or if one single observer counts all sites. However, successive changes in observer identity over time lead to a clear decrease in the ability to reliably estimate a given population trend, and an increase in the number of years of monitoring required to adequately detect the trend.
4. Minimizing temporal changes of observers improve the quality of count data and help taking appropriate management decisions and setting conservation priorities. The same occurs when increasing the number of observers spread over 100 sites. If the population surveyed is composed of few sites, then it is preferable to perform the survey by one observer. In this context, it is important to reconsider how we use estimated population trend values and potentially to scale our decisions according to the direction and duration of estimated trends, instead of setting too precise threshold values before action.

## Graphical abstract

Switching observers impacts wildlife population trend estimates. Are precise thresholds pertinent in conservation if these are out of reach?

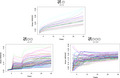

## 1 INTRODUCTION

Conservationists and stakeholders often focus on population dynamics to quantify the scale and significance of ecological and human impacts on wildlife. Estimates of state variables (abundance, occurrence, and species richness, Royle & Dorazio, 2008) and demographic parameters are then critical. Population monitoring programs are therefore designed to quantify the patterns of these variables over time, in order to assess the present status and trends of the populations. National and international programs, such as the IUCN Species Survival Commission Red List Program (IUCN, 2019), U.S. Fish & Wildlife Service (Cowardin & Golet, 1995), or adaptive harvest management programs (Madsen et al., 2017), aim to identify those species most in need of conservation and management attention by using criteria such as quantified reductions in estimated population size (Gärdenfors, 2001; Gärdenfors et al., 2001).

However, wildlife counts only provide estimates and not actual population size, because of various sources of errors such as imperfect detection (Dénes et al., 2015), imperfect abilities to count animals that are detected (Seber, 2002; Thompson, 2002; Williams et al., 2002), misidentification of species or nonexhaustive geographical coverage. When unaccounted for, these errors can introduce considerable estimation bias and obscure important ecological patterns (Wenger & Freeman, 2008), which can reduce the power to detect trends and accuracy of any trends that are detected (Sanz‐Pérez et al., 2020). Integration of systematic sources of count errors into population models can help. Nevertheless, to keep the model simple and avoid overparameterization (principle of parsimony, Vandekerckhove et al., 2015), only a selection of the most important errors should be modeled, according to the situation. In the specific case of highly gregarious species such as waterbirds (Tamisier & Dehorter, 1999), seabirds, and cetaceans (Barlow & Gerrodette, 1996), counts aim to estimate the size of groups of up to several tens of thousands of individuals. In this case, the estimation of group size is particularly likely to be biased and errors must be taken into account. Sources of bias in group size estimation include for instance the count methods, equipment, as well as observer identity. Some studies have focused on measuring this latter source of error. The results collectively suggest that visual estimates of large aggregations of individuals may generally be associated with underestimation combined with high variances within and among observers (Dervieux et al., 1980; Erwin, 1982; Prater, 1979). In these studies, measurement of bias in observer estimation over a range of group sizes is generally evaluated through comparison with aerial photographs (Dervieux et al., 1980; Erwin, 1982; Prater, 1979). However, it is recognized that photographs are far from ideal to assess the true number of individuals in groups, due to other biases that can occur when reading the photo (e.g., definition, movement; Descamps et al., 2011). Studies have therefore used ground counts as a proxy for the "true" number of individuals (Bouché et al., 2012; Smith, 1995), but these are always estimated with a margin of error, and this is particularly the case for high group densities where individuals overlap each other.

Sources of error that change over time, like observer identity, can generate incorrect estimates if they are not properly taken into account (Barker & Sauer, 1992). In particular, it is common over long time series that the staff in charge of counts changed over time. The magnitude of observer differences in estimation error of groups therefore can induce additional variability (Dervieux et al., 1980; Erwin, 1982; Prater, 1979), potentially leading to wrong conclusions regarding trends (McCain et al., 2016). Indeed, the lack of detection of a trend with a given statistical test may correspond to a real absence of a trend or an important type II error (β), hence a lack of statistical power (1−β) which can be due to short time series and/or changes of observers (Gerrodette, 1987; White, 2019). Short time series are potentially misleading: at least 10–20 years of continuous monitoring are generally necessary to achieve a high level of statistical power depending on species, trend strength, and study design (Reynolds et al., 2011; Rueda‐Cediel et al., 2015; White, 2019). However, since both time and resources available for conservation are finite, time series are often shorter than statistically desirable (Field et al. 2007; Hughes et al. 2017). Therefore, managers need to know how much they can trust the apparent trend of a population to reliably identify the sites or species for which conservation action is really needed, and take management decisions (Giron‐Nava et al., 2017; Martin et al., 2012, 2017). Earlier studies have thus examined potentially important trade‐offs between spatial and temporal replication to minimize uncertainty in trend estimates (Rhodes & Jonzén, 2011) and measured the impact of sampling (i.e., count) frequency on trend estimates (Wauchope et al., 2019). However, we are not aware of any study that has assessed the impact of changes in observers, taking into account group estimation error, on the minimum length of population time series (*T*_min_) required to detect significant trends in abundance. This is what we propose to do here, through a simulation study with different scenarios of changes in observers. We also evaluated the effect of observer change on the accuracy (bias and precision) of the trend estimate given the error in estimating group size, with the hypothesis that observer changes reduce confidence in trend estimates.

## 2 METHODS

### 2.1 Population simulation

We simulated a population strictly distributed over 100 sites with initial numbers per site being randomly sampled from a Negative Binomial Distribution with mean *mu* = 300 individuals and the dispersion parameter *size* = 2. Thus, these parameters allow to sample initial group size within the range of values of the 120 group sizes used in the simulation software (see part 2. Group size estimation error). From the initial group size at each site, we simulated the change in “true total population size” (summed over the 100 sites) over time. We considered a simple deterministic model describing density‐independent growth:

(1) $$N_{t+1}=N_{t}\lambda$$

where *N_t_* is population size in year *t*, and λ the population growth rate. Population size in year *t* + 1 depends on population size in year *t,* and the population growth rate remains constant over the entire monitoring period, representing a population evolving in a constant environment. This is an obvious simplification of reality, but not a problem for the present study, which aims at measuring the relative impact of scenarios of changes in observers on *Tmin* required to detect significant trends in abundance, and the accuracy of the trend estimates. The same growth rate was used for all sites. We applied 3 scenarios to the population, that is, declines of 1.1%, 3.9%, or 7.4% per year over 25 years. In this way, we covered most of the thresholds used in conservation programmes: the IUCN Red List applies the criteria of "10% decline over 10 years or 3 generations" (−1.1% per year) to identify Declining species, "30% decline over 10 years or 3 generations" (−3.9% per year) for Vulnerable species, and "50% decline over 10 years or 3 generations" (−7.4% per year) for Endangered species (IUCN, 2019).

### 2.2 Group size estimation error

Group size estimation error data were obtained by an experimental protocol using animal counting simulation software Wildlife Counts (version 2.0.; Hodges, 1993). In this study, 24 experienced observers from the Camargue, Southern France, conducted counting simulation tests on group sizes ranging from 2 to 1,098 individuals with various spatial configurations. The 24 experienced observers sampled in our study are part of several institutions with conservation professionals involved in the counts in Camargue. These professionals have counted in several countries under different field conditions and have to estimate groups with very large numbers of individuals in a relatively short period of time (especially during airplane counts and during ground counts when birds are taking off). Each observer was subjected to 60 tests at average speed (2–21 s to count individuals depending on the size of the groups), and 60 tests at a maximum speed (1–7 s), for a total of 120 tests, identical in group size for all observers. Before each series of 60 tests, two practice tests were performed to prepare the observer. Obviously, display times in the field can be much longer than the times used in the computer tests, but the aim of such tests was only to expose a range of observers to standardized situations, without the aim of measuring their actual observation efficiency in realistic conditions. However, the very short display times for groups of individuals is also a reality in the field, particularly during aerial surveys but also during ground counts to some extent (e.g., when birds are disturbed and take flight).

### 2.3 Simulation of observed population size

**FIGURE 1:**
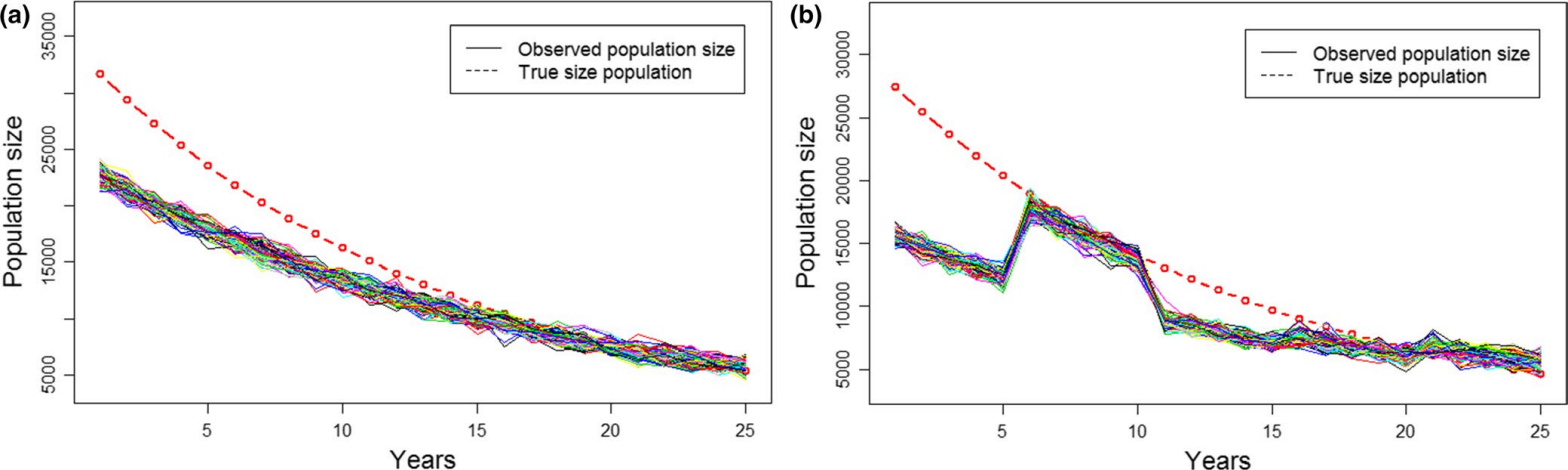
Change in observed and true population size. Population size refers to the total number of individuals over all sites. (a) Example of 100 simulations with random noise of observed population size for one observer in the O1T1 scenario (same observer throughout the study period). In this case sample size *N* = 24 which corresponds to the 24 experienced observers (the 23 other observers are not represented here). (b) Example of one random selection of observers with 100 simulations with random noise of observed population size in the O1T5 scenario (observer changes every 5 years). In this scenario, 99 other random selections were also made (but not represented here), therefore sample size *N* = 100 random selections of observers

Here, the term “observed population size” referred to the sum of count estimates at the 100 sites. We fitted a local polynomial regression (loess regression, Cleveland et al., 1992) for each observer to compute observed group sizes as a function of actual group sizes. For this study, we kept the default parameters of the function, namely a degree of smoothing α = 0.75, type‐2 polynomials and a Gaussian family. We applied the predicted loess regression to the simulated population adding a random noise extracted from the observer specific loess regressions, because a given observer may underestimate or overestimate group size. For the 24 observers, the standard deviation (*SD*) extracted from the loess regression varied from 43 to 103 with an average of 66 ± 18 *SD*. In this way, for each observer, we simulated 100 observed group sizes from each of the 100 sites, over 25 years (Figure 1a; Table 1: scenario O1 T1). Based on these data, five scenarios of changes in observers were run (Table 1). Three gradual scenarios of temporal changes in observers were applied: (T1) the same observer performed the counts for the 25 years of the monitoring; (T5) observers identity changed every five years, that is, 4 observer changes over the entire monitoring period; and (T25) observers identity changed annually, that is, 24 observer changes. Each of the 24 experienced observers was randomly selected, without replacement, to perform monitoring over one year (T25) or for a period of five years (T5). Note that for T25, we randomly re‐selected one of the 24 observers to complete the 25th year of the monitoring.

**TABLE 1:** All scenarios tested for the three simulated true population trends (−1.1% per year, −3.9% per year, −7.4% per year)

Spatial changes (O)	Temporal changes (T)		
	Same observer throughout the study period (T1)	Observers’ identity change every 5 years (T5)	Observers’ identity change every year (T25)
Same observer at all sites (O1)	**O1 T1** (*N* = 24) 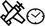	**O1 T5** (*N* = 100) 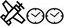	**O1 T25** (*N* = 100) 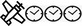
Observers differ for each of the sites (O24)	**O24 T1** (*N* = 100) 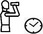	**O24 T5** (*N* = 100) 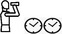	**O24 T25** (*N* = 100) 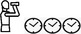

Note

*N* corresponds to the sample size; that is, for all scenarios involving observer changes, 100 random selections of observers were performed. For the scenario where the same observer counted all sites over the entire monitoring period, sample size was 24 which corresponds to the 24 experienced observers who completed the counting simulation tests. The plane icon was used to represent monitoring carried out by one single observer on all sites, as it is often the case during aerial surveys. The observer with binoculars icon was used to represent monitoring carried out by several observers distributed over the sites. Silhouettes reproduced from Flaticon (https://www.flaticon.com/).

**FIGURE 2:**
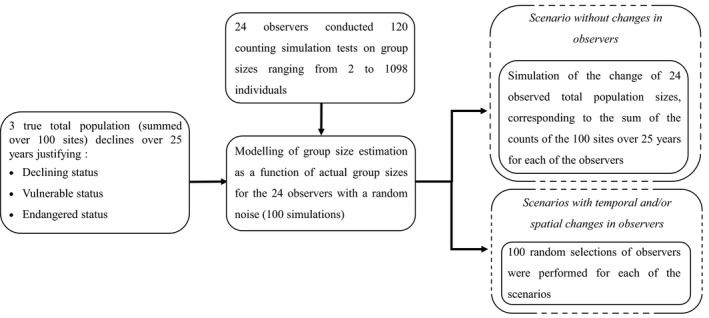
Diagram representing the set of steps used to obtain the observed population sizes for each of the scenarios

Two scenarios of spatial changes in observers were applied: (O1) the same observer performed the counts at all sites; (O24) observers differed at each of the sites. These spatial scenarios may reflect the aerial counts of wildlife (O1), where one person usually carries out the complete survey of many sites (Carretta et al., 2000; Jachmann, 2002). Conversely, (O24) may be more representative of the ground counts or national scheme, with some turnover in nature reserve staff, for example. For O24, the 24 experienced observers were randomly spread over the 100 sites, that is, 20 observers randomly counted four sites and four additional observers randomly counted five sites, respectively. Finally, a total of six scenarios were run for each of the three simulated trends (−1.1%, −3.9% and −7.4% per year, Table 1). In the five scenarios where observers’ identity changed, 100 random selections of observers were performed for each of the scenarios (Figure 1b). The flow diagram in Figure 2 summarizes the different simulation steps.

### 2.4 Power and accuracy analysis

One approach to determining *T*_min_ required to detect significant trends in abundance under the different scenarios of changes in observers is through repetitive simulations (Gerrodette, 1987; Gibbs et al., 1998; White, 2019). Under each scenario, for each random selections of observers, we calculated the proportion of simulations (hereafter statistical power) in which the slope parameter from linear regression was significantly different from 0, at the 0.05 threshold. Although there are many approaches to studying population trends, we used log‐linear regression on the observed population size because this is the simplest and most commonly applied method (Thomas, 1996). The *T*_min_ required to be confident with the detection of a trend in abundance was considered to be obtained when statistical power was equal or greater than 0.8. The significance level 0.05 and statistical power 0.8 were used here as these are historically and commonly used thresholds (Cohen, 1992). When *T*_min_ was greater than 25 years, we set *T*_min_ at 25 years to avoid missing data when compiling the results.

We also evaluated the effect of observer change on the accuracy (bias and precision) of the trend estimate given the error in estimating group size. After checking the normality of the data, we performed *t*‐tests to evaluate the bias of the trend estimate in relation to the simulated theoretical value in the model (1). For example, for a population growth rate of 0.93 (i.e., a trend equal to –7.4% per year), we compared the average of the trend estimates (100 simulations with random noise) for each random selections of observers under the different scenarios to the theoretical value −0.074. To assess the impact of the bias in terms of conservation measures, we had set limits, according to IUCN status, from which the estimated trends are biased. An estimated trend was then considered biased when it leads to a change in conservation status. For example, for the theoretical decline of Endangered species (−7.4%/year), all estimated trends higher than −7.4%/year and lower than −16.4%/year (that corresponds to Critically Endangered species, −80% over 10 years) are biased since the conservation decisions would not be adequate to the actual status of the species.

Normalized root‐mean‐square deviation (NRMSD) was used to measure and compare the precision of the trend estimate between the different scenarios (Hyndman & Koehler, 2006).

$$\text{NRMSD}=\frac{\surd \frac{\sum_{t=1}^{T}{\left({\hat{y}}_{t}‐y_{t}\right)}^{2}}{T}}{\bar{y}}$$

where *ŷ* corresponds to the value predicted by the model in year *t* of monitoring. *y* corresponds to the count done in year *t* of monitoring. *T* is the number of monitoring years, and *ӯ* is the mean of counts done on the number of corresponding monitoring years. NRMSD was calculated for all the number of monitoring years between 3 and 25 years for each of the 100 simulations (random noise) for each random selection of observers under the different scenarios. The lower the NRMSD value, the more precise the trend estimate, because lower values indicate less residual variance.

All analyses were conducted in R (R Core Team, 2017).

## 3 RESULTS

### 3.1 Group size estimation error

The computer exercises showed a frequent underestimation of group size by the observers. On average, they underestimated group sizes by 13% ± 28 (*SD*). Such underestimation was greater when there were more individuals to be counted. The data showed inter‐ and intra‐observer variability in the estimates. For the 24 observers, average individual margin of error ranged from –0.31 (underestimation of 31%) to +0.21 (overestimation of 21%). Only two of the 24 experienced observers showed a persistent tendency to overestimate the counts on average. The average standard deviation of the mean margin of error for the 24 observers was 0.275, ranging from 0.185 to 0.379 (see data in Data Availability Statement).

### 3.2 Effect of observer changes on the *T*_min_ required to detect significant trends

**FIGURE 3:**
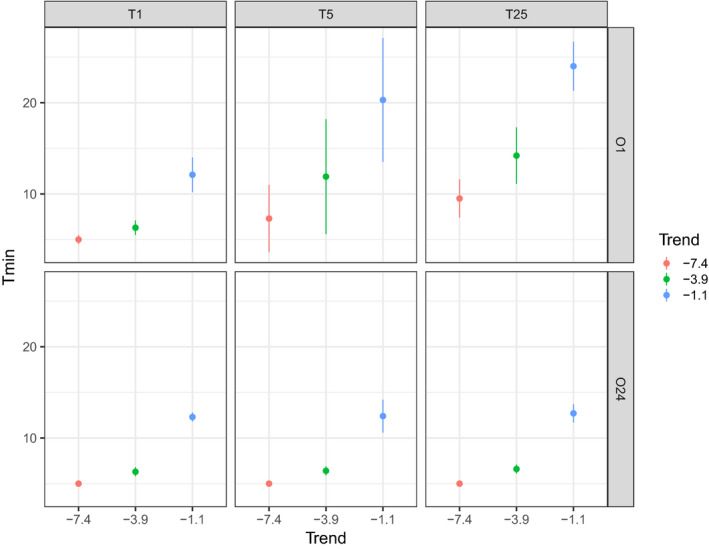
Minimum time required (in years) to detect significant trends in abundance (mean *T*_min_ ± *SD*) under the different scenarios of changes in observers

For each of the scenarios, *T*_min_ results show that steeper decreasing trends required less time to be detected (Figure 3). Temporal changes in observers influenced the number of years required to detect a trend when the same observer counted all sites in a given year (scenarios O1, Figure 3). The gradual increase in *T*_min_ values under the various O1 scenarios was the consequence of additional variability in the counting data due to more frequent temporal changes of observers. However, *T*_min_ remained similar to that of scenario O1 T1 when observer changes occurred spatially (scenario O24 T1) and both spatially and temporally (scenarios O24 T5 and O24 T25; Figure 3). In this configuration (20 observers randomly counted 4 sites and 4 additional observers randomly counted 5 sites), observer spatial changes reduced the influence of observer temporal changes on *T*_min_ values. In scenario O1 T1, the differences in the capacity to estimate group size (values of random noise extract from the observer specific loess regressions, see Method section 3) among observers did not have a strong impact on the standard deviation. In this scenario, for the example of a trend of –7.4% per year, the observers needed 6 years to detect the trend when the random noise value was the highest, because of greater variability of the counts. For scenarios where observer changes occurred only temporally, the wide standard deviation highlighted the high variability of *T*_min_ values induced by the 100 random selections of observers (Figure 3). Changes of observers every five years (O1 T5) induced greater variability in *T*_min_ values compared to an annual change of observer (O1 T25; Figure 3).

### 3.3 Effect of observer changes on the accuracy (bias and precision) of the trend estimate

#### 3.3.1 Trend bias analysis

**FIGURE 4:**
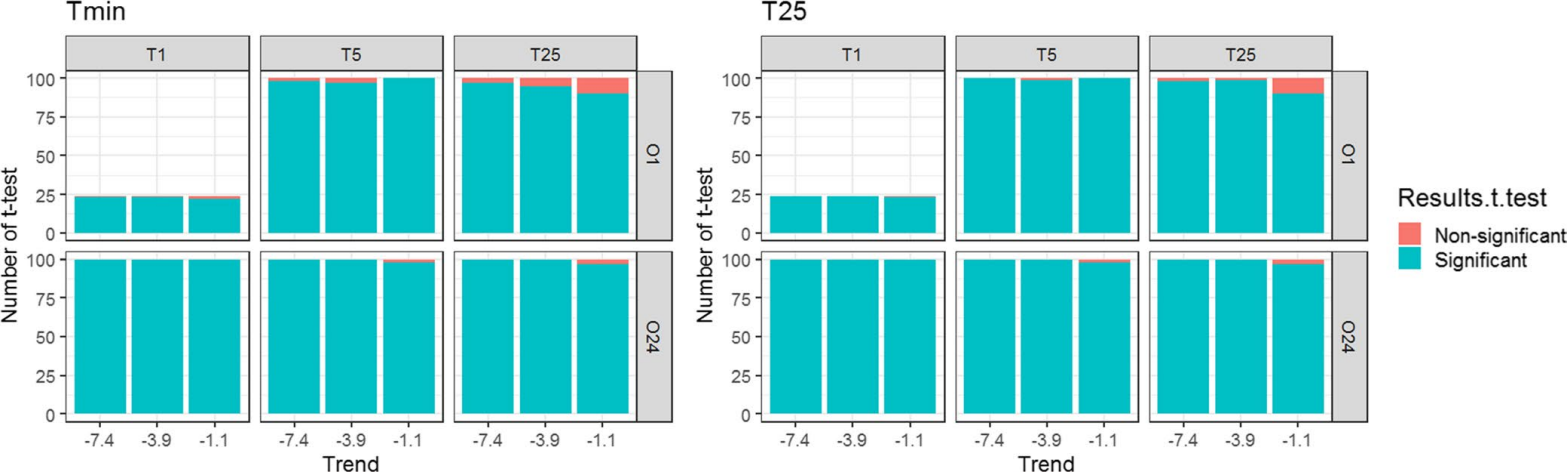
*t*‐test results for *T*_min_ and 25 years of monitoring. The results are expressed as the number of nonsignificant (detection of the correct estimation of the actual trend value) and significant tests. Tests compared the average of the trend estimates (100 simulations with random noise) of each of the samples of the different scenarios to the actual values of trends

**FIGURE 5:**
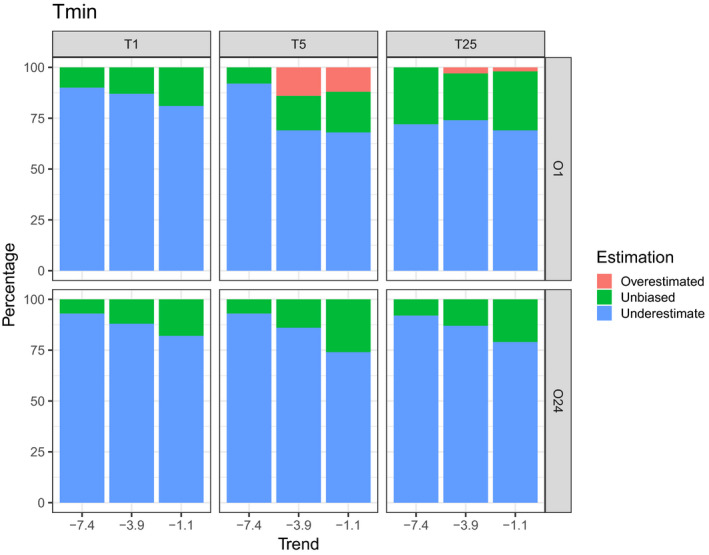
Plot representing the percentage of overestimated, unbiased, and underestimated trends for all scenarios and trends tested. These results are extracted from *T*_min_ to detect significant trends

**FIGURE 6:**
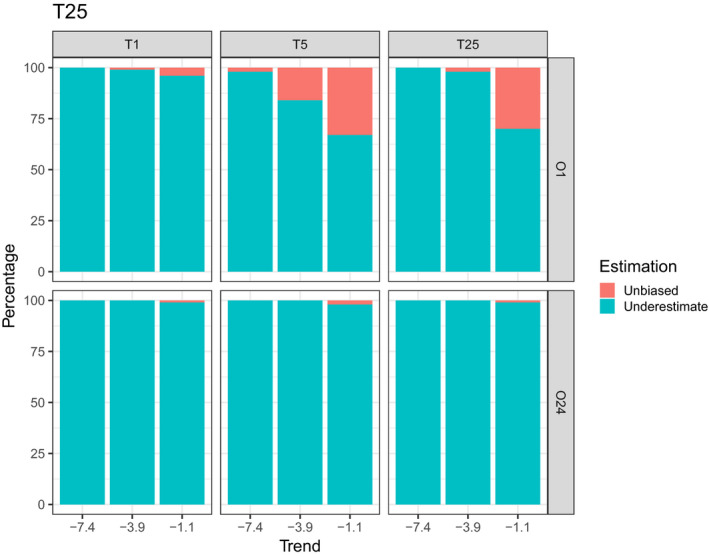
Plot representing the percentage of overestimated, unbiased, and underestimated trends for all scenarios and trends tested. These results are extracted from 25 years of monitoring

In general, for all scenarios and trends, observers did not appear to be able to accurately estimate the actual rate of change in population size. However, the direction of the trend was detected in 94% of all cases considered (Table S1). Figure 4 shows the results of the *t*‐tests for *T*_min_ and for 25 years of monitoring, respectively. The proportion of trends accurately detected was extremely low for each of the scenarios and trends (Figure 4). For all trends and all scenarios combined, 2% of simulations accurately detected the trends for *T*_min_ and 0.8% of simulations accurately detected the trends for 25 years of monitoring. In addition, according to IUCN status, for all trends and all scenarios combined, 16.6% of simulations are unbiased (81.3% underestimates and 2.1% overestimated the actual trend value) for *T*_min_ (Figure 5) and 5.8% of simulations are unbiased (94.2% underestimates the actual trend value) for 25 years of monitoring (Figure 6).

#### 3.3.2 Trend precision analysis

**FIGURE 7:**
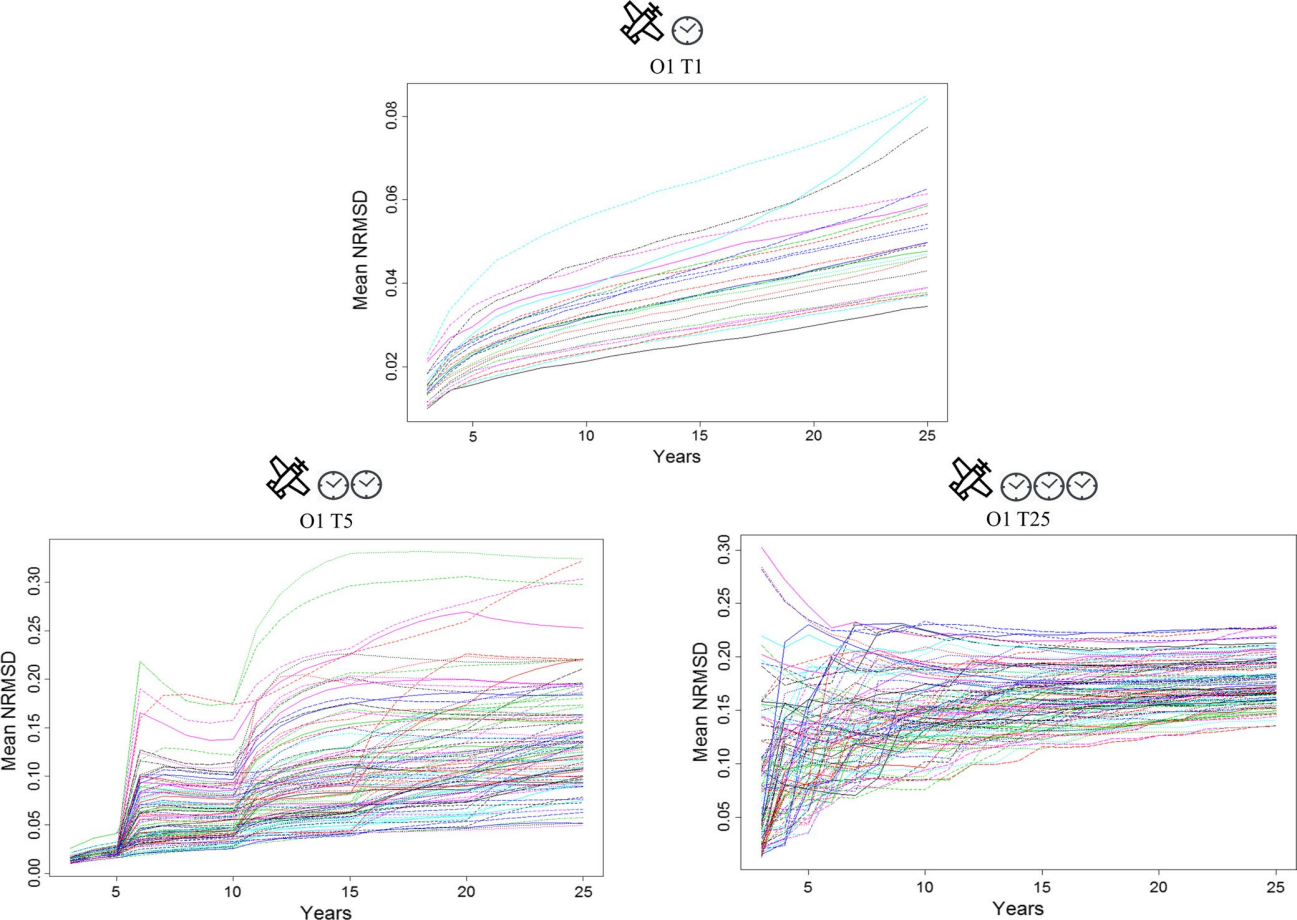
Mean NRMSD according to the number of years of monitoring for the trend – 7.4% per year and for all scenarios with only temporal changes in observers (100 simulations with random noise for each of the 24 observers for the scenario O1 T1 and for each of the 100 random selections of observers for scenarios O1 T5 and O1 T25)

Similar values of NRMSD were obtained for a given scenario, regardless of the trends tested. For example, for scenarios O1 T1, initial mean values of NRMSD were between 0.01 and 0.025 for each of the trends and increased up to 25 years of monitoring to reach mean values between 0.02 and 0.08 (Figure S3). In this scenario, the increase of NRMSD over the time (Figure 1, 7, O1 T1) reflected the increasing residual variance induced by variations in the counting abilities of the 24 experienced observers (values of random noise extracted from the observer specific loess regressions, see Method section 3).

On the other hand, changing the identity of observers over time (scenarios O1 T5 and O1 T25, Figure 7) led to a decrease in the precision of the slope parameter estimate. Mean NRMSD values increased (greater residual variance) as temporal changes became more frequent. For example, for a trend of −7.4% per year, we obtained: (a) Scenario O1 T1: mean NRMSD between 0.01 and 0.04 for 5 years of monitoring (the average *T*_min_); (b) Scenario O1 T5: mean NRMSD between 0.02 and 0.19 for 7 years of monitoring (the average *T*_min_); and (3) Scenario O1 T25: mean NRMSD between 0.07 and 0.23 for 10 years of monitoring (the average *T*_min_) (Figure 7). The differences between the scenarios became clearer if the slope parameters were estimated over 25 years of monitoring. Very frequent changes of observers, such as in scenario O1 T25, induced high average NRMSD values (range 0.13–0.23) with a smaller amplitude than in scenario O1 T5 (range 0.03–0.34) (Figure 7). When observers’ identity changed every fifth year, random draws of observers could induce low mean NRMSD values (Figure 7). However, the majority of the values remained high: 76% of the values were between 0.10 and 0.34 (Figure 7).

When observers’ identity changed spatially (scenarios O24 T1, O24 T5, O24 T25), the temporal changes did not influence the NRMSD mean values, regardless of the trend value (Figure S4).

## 4 DISCUSSION

This study shows that under realistic field conditions where observers’ identity changes temporally and spatially, wildlife population size and trend estimates are similar if many observers are spatially distributed between the different sites, or if one single observer counts all sites. Our results also show that successive changes in observer identity over time reduce our ability to precisely estimate a given population trend and increase the number of years of monitoring required to adequately detect the trend.

In our study, the counting data were analyzed by log‐linear regression and do not include statistical practices that adjust for variation among observers such as random‐effect intercepts for individual observers (Link & Sauer, 1997). Techniques that fail to take into account the variation between observers remain popular among monitoring programs (Klvaňová & Voříšek 2007; Rosenstock et al., 2002). Indeed, the analysis of data that allows accounting for variations in inter‐observer group size estimates is not always straightforward, and coordinators of monitoring programs are sometimes skeptical about accounting for it because they find the analyses too complex. One example is the Pan‐European Common Bird Monitoring Scheme (PECBMS), which produces national and supranational indexes by using the TRIM software (TRends and Indices for Monitoring data; Klvaňová & Voříšek, 2007). TRIM is also used to assess conservation status for IUCN Red List assessments (Criterion A; Maes et al., 2015). While frequent changes in observers are inherent to monitoring, this study advocates for considering the identity of the observers in order to take into account the variations in inter‐observer group size estimates during the statistical analysis.

Our study highlighted a general trend for observers to underestimate group size during counts (as found by Dervieux et al., 1980; Erwin, 1982; Prater, 1979), and their difficulty in precisely detecting the value of the trend (in this study the theoretical trend is detected in 1.3% of the cases). The computer tests also showed a wide variation in count ability among the observers, even though all of them were experienced fieldworkers. This observation is in line with studies on human counting capacities (Erwin, 1982; Prater, 1979). The ex situ protocol used in this study was however novel in that it allowed us to focus only on the intrinsic ability of observers to estimate group size, while standardizing other regular sources of group count errors such as habitat type, weather conditions, species behavior, time, and equipment available (Barker & Sauer, 1992) which may confound similar studies conducted on field data.

Our simulations show that keeping the same observer over time did not remove bias in the detection of a trend value. For all the scenarios tested in this study, observers performed very poorly at detecting the theoretical value of the population trend, regardless of the steepness of the trend tested and the number of years of monitoring. Trends tested in this study correspond to indicators commonly used in national and international monitoring programs (e.g., 30% decline over 10 years or three generations to classify species as having a Vulnerable status, Hearn et al., 2018; IUCN, 2019). Given these results, such threshold values used to assess the conservation status of a species or a population seem inappropriate when relying on counting data of highly gregarious species such as birds, in view of the biases caused by group size estimation error. Whether underestimated or overestimated compared to the real value, misestimating trends in population size is costly, either ecologically if this causes inaction where it would be desirable (in the case of underestimation of population declines) or financially if overestimation of population declines leads to conservation intervention where it is not a priority.

Although the estimated trend values remain highly biased, our results show that the gap between estimated and real population sizes decreased over longer monitoring periods, in the case of declining populations. This did not simply reflect a gradual improvement in observer accuracy over time, which could arise through learning (Garel et al., 2005; Williams et al., 2006), since this was not taken into account in our simulations. At the beginning of the counts when the population was still relatively large, there was a wide gap between estimated and actual population sizes, which decreased as the population gradually declined following the negative trends we used. Indeed, such improvement in the quality of the counts was due to the fact that, on average, observers tended to underestimate smaller group sizes to a lower extent (for group sizes between 2 and 201 individuals observers underestimated by 0.34% ± 10.5 (*SD*); for group sizes between 208 and 1,098 individuals observers underestimated by 26.4% ± 13.0 (*SD*)). Similarly, trend estimates would become increasingly underestimated over time in the case of a population increase.

In addition to the effect of group size, the number of years of monitoring appeared to improve trend precision (Supporting 2), although this occurred through a greater precision rather than a less biased mean estimated value (see also Yates, 1953). Some studies highlight that longer time series are needed to obtain smaller confidence intervals, and to detect a decline when it is of low magnitude, that is, −1% per year (Connors et al., 2014; Tománková et al., 2013; Wauchope et al., 2019; Wilson et al., 2011). Accordingly, we found that it is most challenging to detect the direction of a slight trend (sensu Wauchope et al., 2019) when temporal changes in observers occur (Table S1). Thus, for a large decrease in an initially abundant population, counts unadjusted for error in estimating groups may still allow detection of the direction of the trend, although not the precise value of the trend itself, even in the context of frequent changes of observers in time series as is often the case in the field (example of the Common Pochard, Folliot, 2018).

Our results show that having different observers counting different sites did not induce overall losses of statistical power and precision of estimated trends compared to a theoretical ideal situation where one single observer would count all sites. When the number of sites is large, the large number of observers allows for a high variability of estimation capabilities that actually buffer each other, leading to a lower uncertainty in the estimation of the trend. If the number of sites monitored remains large and the number of observers decreases, that is, fewer observers count more sites each, more time is needed to detect the trend (2 observers each monitoring 50 sites: mean *T*_min_: 7 ± 1 (*SD*) years, which is on average two year longer than when 24 observers are spatially distributed between 100 sites) and a loss of precision of estimated trends is induced in some cases (Figure S5). This pattern likely arises because there are fewer individual processes to buffer each other, so that the poor abilities of one given observer are more likely to lead to biased overall results. In addition to this, when counts are carried out over smaller areas where fewer sites are counted, such as the 40 sites counted from the ground in Camargue nature reserves, in Southern France (Tamisier & Dehorter, 1999), spatial changes in observers decrease confidence in the estimation of derived trends (24 observers monitor each one site: mean *T*_min_: 6 ± 1 (*SD*) years, which is on average one year longer than when 24 observers are spatially distributed over 100 sites and a loss of precision of estimated trends is induced, see Figure S6). In this context, it is preferable to favor a single observer for all sites where only a few sites need to be sampled frequently, especially if spatial autocorrelation in population dynamics is high (Rhodes & Jonzén, 2011).

To achieve an optimal balance between cost‐effectiveness and precision of wildlife monitoring programmes, our study showed that the most important thing to avoid is temporal change in observer identity. Indeed, a greater frequency of observer change gradually increased the period necessary to detect a significant trend, and decreased the precision of estimated trends for a given monitoring duration, although the direction of the trend was generally adequately detected (See also supporting 1). Wildlife management however depends on long‐term monitoring databases, especially so for species with longer generation times (White, 2019). The collection of such data can be difficult, expensive, and labor‐intensive (Williams et al., 2002). Consequently, many monitoring programs often require the use of a large number of observers, both instantaneously to cover the many sites used by the animal population, and over time as people change job or retire (Schwarz & Seber, 1999), which introduces an additional source of variability into observations. The results of this study therefore first call for sufficient and sustained funding of monitoring schemes, so that staff can receive the appropriate similar qualification and remain involved in monitoring for prolonged periods, instead of relying on successive volunteers of very unequal count abilities. Equally, our results suggest that long time series can help to compensate for some of the biases introduced by changing observer identity. In such cases, interpretation of short‐term monitoring (e.g., 3–5 years for birds species) could be highly misleading. Ultimately, these results can help to design future counting protocols with the aim of finding the best compromise between high precision (minimizing temporal changes in observers to maximize the precision of the trend estimate), cost‐effectiveness (minimizing temporal changes of observers to achieve high statistical power and decrease the monitoring period), and logistic feasibility (temporal changes of observers are inherent to population monitoring). This compromise must be adapted to the species in question (Ficetola et al., 2018), for example owing to its gregariousness and consequent difficulty to be counted, to the management objectives (Lindenmayer & Likens, 2009; McDonald‐Madden et al., 2010) and adapted to time period to match those used in conservation schemes, such as IUCN criteria, while achieving high statistical power (White, 2019).

## 5 CONCLUSIONS

In order to make the right management decisions, population trend analysis should be based on the highest possible quality of count data. Building a count protocol adapted to conservation objectives, minimizing temporal changes of observers trying to maintain staff positions, and considering the importance of the number of observers distributed spatially according to the number of sites monitored all improve the quality of count data. In addition, it is important to reconsider how we use estimated population trend values, and potentially to base our decisions on the direction and duration of estimated trends without requiring these to cross predefined, precise thresholds. Ensuring that we collect reliable count data will provide help taking appropriate management decisions and setting conservation priorities in this context. Alternative methods based on imagery have been gaining ground over the last decades (Akçay et al., 2020; Hodgson et al., 2018; Lyons et al., 2019), and more particularly with automated computer vision software (Chabot & Francis, 2016; Hollings et al., 2018). However, computer vision software may work under some particular conditions but they are generally biased and known to fail in several situations (Chabot & Francis, 2016; Hollings et al., 2018) even if considerable improvements are underway (González‐Villa & Cruz, 2019). We expect that the continuing technological developments (sophisticated image analysis software and advances in camera and drone technology) in the analysis of remotely sensed data will control the error in estimating groups’ size in many more situations than at present, where observers remain the most widely used means to monitor animal populations.

## CONFLICT OF INTEREST

The authors declare this research was undertaken in absence of any conflict of interest.

## AUTHOR CONTRIBUTIONS

**David Vallecillo:** Conceptualization (equal); Data curation (equal); Formal analysis (equal); Methodology (equal); Software (equal); Validation (equal); Writing‐original draft (lead); Writing‐review & editing (equal). **Michel Gauthier‐Clerc:** Conceptualization (equal); Data curation (equal); Funding acquisition (lead); Investigation (equal); Methodology (equal); Project administration (equal); Resources (equal); Validation (equal); Writing‐original draft (supporting). **Matthieu Guillemain:** Conceptualization (equal); Methodology (equal); Project administration (equal); Supervision (equal); Validation (equal); Writing‐original draft (lead); Writing‐review & editing (equal). **Marion Vittecoq:** Conceptualization (equal); Formal analysis (equal); Methodology (equal); Validation (equal); Writing‐original draft (supporting). **Philippe Vandewalle:** Conceptualization (equal); Data curation (equal); Investigation (equal); Validation (equal); Writing‐original draft (supporting). **Benjamin Roche:** Conceptualization (equal); Formal analysis (equal); Methodology (equal); Validation (equal); Writing‐original draft (supporting). **Jocelyn Champagnon:** Conceptualization (equal); Funding acquisition (equal); Methodology (equal); Project administration (equal); Resources (equal); Supervision (equal); Validation (equal); Writing‐original draft (lead); Writing‐review & editing (equal).

## Supporting information

Supplementary Material

## Data Availability

All data used in our analysis are available from https://doi.org/10.6084/m9.figshare.13498524.v1.

## References

[ece37191-bib-0001] Akçay, H. G. , Kabasakal, B. , Aksu, D. , Demir, N. , Öz, M. , & Erdoğan, A. (2020). Automated bird counting with deep learning for regional bird distribution mapping. Animals, 10(7), 1207. 10.3390/ani10071207 PMC7401518 32708550

[ece37191-bib-0002] Barker, R. J. , & Sauer, J. R. (1992). Modelling population change from time series data. In D. R. McCullough , & R. H. Barrett (Eds.), Wildlife 2001: Populations (pp. 182–194). Springer Netherlands.

[ece37191-bib-0003] Barlow, J. , & Gerrodette, T. (1996). Abundance of cetaceans in California waters based on 1991 and 1993 ship surveys.

[ece37191-bib-0004] Bouché, P. , Lejeune, P. , & Vermeulen, C. (2012). How to count elephants in West African savannahs? Synthesis and comparison of main gamecount methods. Biotechnologie, Agronomie, Société et Environnement, 16(1), 77–91.

[ece37191-bib-0005] Carretta, J. V. , Lowry, M. S. , Stinchcomb, C. , Lynn, M. S. , & Cosgrove, R. E. (2000). Distribution and abundance of marine mammals at San Clemente Island and surrounding offshore waters: Results from aerial and ground surveys in 1998 and 1999.

[ece37191-bib-0061] Chabot D. , Francis C. M. (2016). Computer‐automated bird detection and counts in high‐resolution aerial images: a review. Journal of Field Ornithology, 87(4), 343–359. 10.1111/jofo.12171

[ece37191-bib-0007] Cleveland, W. S. , Grosse, E. , & Shyu, W. M. (1992). Local regression models. Chapter 8. In J. M. Chambers , & T. J. Hastie (Eds.), Statistical models in S (608 p.). Wadsworth & Brooks/Cole.

[ece37191-bib-0008] Cohen, J. (1992). A power primer. Psychological Bulletin, 112(1), 155. 10.1037/0033-2909.112.1.155 19565683

[ece37191-bib-0009] Connors, B. M. , Cooper, A. B. , Peterman, R. M. , & Dulvy, N. K. (2014). The false classification of extinction risk in noisy environments. Proceedings of the Royal Society B: Biological Sciences, 281(1787), 20132935. 10.1098/rspb.2013.2935 PMC4071530 24898368

[ece37191-bib-0010] Cowardin, L. M. , & Golet, F. C. (1995). US Fish and Wildlife Service 1979 wetland classification: A review. Vegetatio, 118(1–2), 139–152. 10.1007/BF00045196

[ece37191-bib-0011] Dénes, F. V. , Silveira, L. F. , & Beissinger, S. R. (2015). Estimating abundance of unmarked animal populations: Accounting for imperfect detection and other sources of zero inflation. Methods in Ecology and Evolution, 6(5), 543–556. 10.1111/2041-210X.12333

[ece37191-bib-0012] Dervieux, A. , Lebreton, J.‐D. , & Tamisier, A. (1980). Technique et fiabilité des dénombrements aériens de canards et de foulques hivernant en Camargue. Revue D’ecologie (La Terre Et La Vie), 34(1), 69–99.

[ece37191-bib-0013] Descamps, S. , Béchet, A. , Descombes, X. , Arnaud, A. , & Zerubia, J. (2011). An automatic counter for aerial images of aggregations of large birds. Bird Study, 58(3), 302–308. 10.1080/00063657.2011.588195

[ece37191-bib-0014] Erwin, R. M. (1982). Observer variability in estimating numbers: An experiment. Journal of Field Ornithology, 53(2), 159–167.

[ece37191-bib-0015] Ficetola, G. F. , Romano, A. , Salvidio, S. , & Sindaco, R. (2018). Optimizing monitoring schemes to detect trends in abundance over broad scales. Animal Conservation, 21(3), 221–231. 10.1111/acv.12356

[ece37191-bib-0065] Field S. A. , O'Connor P. J. , Tyre A. J. , Possingham H. P. (2007). Making monitoring meaningful. Austral Ecology, 32(5), 485–491. 10.1111/j.1442-9993.2007.01715.x

[ece37191-bib-0016] Folliot, B. (2018, December 17). Dynamique des espèces exploitées: le cas du fuligule milouin (Aythya ferina) dans le Paléarctique (thesis). Retrieved from http://www.theses.fr/2018MONTG043

[ece37191-bib-0017] Gärdenfors, U. (2001). Classifying threatened species at national versus global levels. Trends in Ecology & Evolution, 16(9), 511–516. 10.1016/S0169-5347(01)02214-5

[ece37191-bib-0018] Gärdenfors, U. , Hilton‐Taylor, C. , Mace, G. M. , & Rodríguez, J. P. (2001). The application of IUCN red list criteria at regional levels. Conservation Biology, 15(5), 1206–1212. 10.1111/j.1523-1739.2001.00112.x

[ece37191-bib-0019] Garel, M. , Cugnasse, J.‐M. , Gaillard, J.‐M. , Loison, A. , Santosa, Y. , & Maublanc, M.‐L. (2005). Effect of observer experience on the monitoring of a mouflon population. Acta Theriologica, 50(1), 109–114. 10.1007/BF03192623

[ece37191-bib-0020] Gerrodette, T. (1987). A power analysis for detecting trends. Ecology, 68(5), 1364–1372. 10.2307/1939220

[ece37191-bib-0021] Gibbs, J. P. , Droege, S. , & Eagle, P. (1998). Monitoring populations of plants and animals. BioScience, 48(11), 935–940. 10.2307/1313297

[ece37191-bib-0022] Giron‐Nava, A. , James, C. C. , Johnson, A. F. , Dannecker, D. , Kolody, B. , Lee, A. , Nagarkar, M. , Pao, G. M. , Ye, H. , Johns, D. G. , & Sugihara, G. (2017). Quantitative argument for long‐term ecological monitoring. Marine Ecology Progress Series, 572, 269–274. 10.3354/meps12149

[ece37191-bib-0023] González‐Villa, J. , & Cruz, M. (2019). The CountEm software: Simple, efficient and unbiased population size estimation. Ecography, 43(2), 251–255. 10.1111/ecog.04862

[ece37191-bib-0024] Hearn, R. , Nagy, S. , van Roomen, M. , Hall, C. , Citegese, G. , Donald, P. , Hagemeijer, W. , Langendoen, T. (2018). Guidelines on waterbird monitoring. AEWA Conservation Guidelines No. 9. AEWA Technical Series No. XX. Bonn, Germany.

[ece37191-bib-0025] Hodges, J. I. (1993). COUNT: A simulation for learning to estimate wildlife numbers. Wildlife Society Bulletin (1973–2006), 21(1), 96–97.

[ece37191-bib-0026] Hodgson, J. C. , Mott, R. , Baylis, S. M. , Pham, T. T. , Wotherspoon, S. , Kilpatrick, A. D. , Raja Segaran, R. , Reid, I. , Terauds, A. , & Koh, L. P. (2018). Drones count wildlife more accurately and precisely than humans. Methods in Ecology and Evolution, 9(5), 1160–1167. 10.1111/2041-210X.12974

[ece37191-bib-0059] Hollings T. , Burgman M. , van Andel M. , Gilbert M. , Robinson T. , Robinson A. (2018). How do you find the green sheep? A critical review of the use of remotely sensed imagery to detect and count animals. Methods in Ecology and Evolution, 9(4), 881–892. 10.1111/2041-210x.12973

[ece37191-bib-0060] Hughes B. B. , Beas‐Luna R. , Barner A. K. , Brewitt K. , Brumbaugh D. R. , Cerny‐Chipman E. B. , Close S. L. , Coblentz K. E. , de Nesnera K. L. , Drobnitch S. T. , Figurski J. D. , Focht B. , Friedman M. , Freiwald J. , Heady K. K. , Heady W. N. , Hettinger A. , Johnson A. , Karr K. A. , … & Carr Mark H. (2017). Long‐Term Studies Contribute Disproportionately to Ecology and Policy. BioScience, 67(3), 271–281. 10.1093/biosci/biw185

[ece37191-bib-0027] Hyndman, R. J. , & Koehler, A. B. (2006). Another look at measures of forecast accuracy. International Journal of Forecasting, 22(4), 679–688. 10.1016/j.ijforecast.2006.03.001

[ece37191-bib-0028] IUCN . (2019). The IUCN red list of threatened species. Version 2019‐1. Retrieved from http://www.iucnredlist.org

[ece37191-bib-0029] Jachmann, H. (2002). Comparison of aerial counts with ground counts for large African herbivores. Journal of Applied Ecology, 39(5), 841–852. 10.1046/j.1365-2664.2002.00752.x

[ece37191-bib-0063] Klvaňová, A. , & Voříšek, P. (2007). Review on large‐scale generic population monitoring schemes in Europe 2007. Bird Census News, 20(2), 50–56.

[ece37191-bib-0030] Lindenmayer, D. B. , & Likens, G. E. (2009). Adaptive monitoring: A new paradigm for long‐term research and monitoring. Trends in Ecology & Evolution, 24(9), 482–486. 10.1016/j.tree.2009.03.005 19409648

[ece37191-bib-0064] Link W. A. , Sauer J. R. (1997). Estimation of Population Trajectories from Count Data. Biometrics, 53(2), 488. 10.2307/2533952

[ece37191-bib-0031] Lyons, M. B. , Brandis, K. J. , Murray, N. J. , Wilshire, J. H. , McCann, J. A. , Kingsford, R. T. , & Callaghan, C. T. (2019). Monitoring large and complex wildlife aggregations with drones. Methods in Ecology and Evolution, 10(7), 1024–1035. 10.1111/2041-210X.13194

[ece37191-bib-0066] Maes D. , Isaac N. J. B. , Harrower C. A. , Collen B. , van Strien A. J. , Roy D. B. (2015). The use of opportunistic data for IUCN Red List assessments. Biological Journal of the Linnean Society, 115(3), 690–706. 10.1111/bij.12530

[ece37191-bib-0032] Madsen, J. , Williams, J. H. , Johnson, F. A. , Tombre, I. M. , Dereliev, S. , & Kuijken, E. (2017). Implementation of the first adaptive management plan for a European migratory waterbird population: The case of the Svalbard pink‐footed goose *Anser brachyrhynchus* . Ambio, 46(S2), 275–289. 10.1007/s13280-016-0888-0 28215011 PMC5316328

[ece37191-bib-0033] Martin, T. G. , Camaclang, A. E. , Possingham, H. P. , Maguire, L. A. , & Chadès, I. (2017). Timing of protection of critical habitat matters. Conservation Letters, 10(3), 308–316. 10.1111/conl.12266

[ece37191-bib-0034] Martin, T. G. , Nally, S. , Burbidge, A. A. , Arnall, S. , Garnett, S. T. , Hayward, M. W. , Lumsden, L. F. , Menkhorst, P. , McDonald‐Madden, E. , & Possingham, H. P. (2012). Acting fast helps avoid extinction: Acting fast avoids extinctions. Conservation Letters, 5(4), 274–280. 10.1111/j.1755-263X.2012.00239.x

[ece37191-bib-0035] McCain, C. , Szewczyk, T. , & Bracy Knight, K. (2016). Population variability complicates the accurate detection of climate change responses. Global Change Biology, 22(6), 2081–2093. 10.1111/gcb.13211 26725404

[ece37191-bib-0036] McDonald‐Madden, E. , Baxter, P. W. , Fuller, R. A. , Martin, T. G. , Game, E. T. , Montambault, J. , & Possingham, H. P. (2010). Monitoring does not always count. Trends in Ecology & Evolution, 25(10), 547–550. 10.1016/j.tree.2010.07.002 20727614

[ece37191-bib-0037] Prater, A. J. (1979). Trends in accuracy of counting birds. Bird Study, 26(3), 198–200.

[ece37191-bib-0038] R Core Team . (2017). R: A language and environment for statistical computing. R Foundation for Statistical Computing.

[ece37191-bib-0039] Reynolds, J. H. , Thompson, W. L. , & Russell, B. (2011). Planning for success: Identifying effective and efficient survey designs for monitoring. Biological Conservation, 144(5), 1278–1284. 10.1016/j.biocon.2010.12.002

[ece37191-bib-0040] Rhodes, J. R. , & Jonzén, N. (2011). Monitoring temporal trends in spatially structured populations: How should sampling effort be allocated between space and time? Ecography, 34(6), 1040–1048. 10.1111/j.1600-0587.2011.06370.x

[ece37191-bib-0062] Rosenstock S. S. , Anderson D. R. , Giesen K. M. , Leukering T. , Carter M. F. (2002). Landbird Counting Techniques: Current Practices and an Alternative. The Auk, 119(1), 46–53. 10.1093/auk/119.1.46

[ece37191-bib-0041] Royle, J. A. , & Dorazio, R. M. (2008). Hierarchical modeling and inference in ecology: The analysis of data from populations, metapopulations and communities. Elsevier.

[ece37191-bib-0042] Rueda‐Cediel, P. , Anderson, K. E. , Regan, T. J. , Franklin, J. , & Regan, H. M. (2015). Combined influences of model choice, data quality, and data quantity when estimating population trends. PLoS One, 10(7), e0132255. 10.1371/journal.pone.0132255 26177511 PMC4503393

[ece37191-bib-0043] Sanz‐Pérez, A. , Sollmann, R. , Sardà‐Palomera, F. , Bota, G. , & Giralt, D. (2020). The role of detectability on bird population trend estimates in an open farmland landscape. Biodiversity and Conservation, 29(6), 1747–1765. 10.1007/s10531-020-01948-0

[ece37191-bib-0044] Schwarz, C. J. , & Seber, G. A. (1999). Estimating animal abundance: Review III. Statistical Science, 14, 427–456.

[ece37191-bib-0045] Seber, G. A. (2002). The estimation of animal abundance and related parameters. Blackburn Press. xvii.

[ece37191-bib-0046] Smith, G. W. (1995). A critical review of the aerial and ground surveys of breeding waterfowl in North America. (No. FWS‐SR‐5). Fish and Wildlife Service Laurel MD Office of Migratory Bird Management. Retrieved from http://www.dtic.mil/docs/citations/ADA322667

[ece37191-bib-0047] Tamisier, A. , & Dehorter, O. (1999). Camargue, canards et foulques: fonctionnement et devenir d’un prestigieux quartier d’hiver. Centre ornithologique du Gard.

[ece37191-bib-0048] Thomas, L. (1996). Monitoring long‐term population change: Why are there so many analysis methods? Ecology, 77(1), 49–58. 10.2307/2265653

[ece37191-bib-0049] Thompson, W. L. (2002). Towards reliable bird surveys: Accounting for individuals present but not detected. The Auk, 119(1), 18–25. 10.1093/auk/119.1.18

[ece37191-bib-0050] Tománková, I. , Boland, H. , Reid, N. , & Fox, A. D. (2013). Assessing the extent to which temporal changes in waterbird community composition are driven by either local, regional or global factors. Aquatic Conservation: Marine and Freshwater Ecosystems, 23(2), 343–355. 10.1002/aqc.2303

[ece37191-bib-0051] Vandekerckhove, J. , Matzke, D. , & Wagenmakers, E.‐J. (2015). In J. R. Busemeyer , Z. Wang , J. T. Townsend , & A. Eidels (Eds.), Model comparison and the principle of parsimony (Vol. 1). Oxford University Press.

[ece37191-bib-0052] Wauchope, H. , Amano, T. , Sutherland, W. , & Johnston, A. (2019). When can we trust population trends? A method for quantifying the effects of sampling interval and duration. Methods in Ecology and Evolution, 10(12), 2067–2078. 10.1111/2041-210X.13302

[ece37191-bib-0053] Wenger, S. J. , & Freeman, M. C. (2008). Estimating species occurrence, abundance, and detection probability using zero‐inflated distributions. Ecology, 89(10), 2953–2959. 10.1890/07-1127.1 18959332

[ece37191-bib-0054] White, E. R. (2019). Minimum time required to detect population trends: The need for long‐term monitoring programs. BioScience, 69(1), 40–46. 10.1093/biosci/biy144

[ece37191-bib-0055] Williams, B. K. , Nichols, J. D. , & Conroy, M. J. (2002). Analysis and management of animal populations. Academic Press.

[ece37191-bib-0056] Williams, I. D. , Walsh, W. J. , Tissot, B. N. , & Hallacher, L. E. (2006). Impact of observers’ experience level on counts of fishes in underwater visual surveys. Marine Ecology Progress Series, 310, 185–191. 10.3354/meps310185

[ece37191-bib-0057] Wilson, H. B. , Kendall, B. E. , & Possingham, H. P. (2011). Variability in population abundance and the classification of extinction risk. Conservation Biology, 25(4), 747–757. 10.1111/j.1523-1739.2011.01671.x 21480994

[ece37191-bib-0058] Yates, F. (1953). Sampling methods for censuses and surveys (2nd ed.). London: Charles Griffin & Co., Ltd.

