# Supplementary material for: Reliability of animal counts and implications for the interpretation of trends
Source: Ecol Evol. 2021 Jan 27;11(5):2249–60. doi: 10.1002/ece3.7191 (PMC7920765; doi:10.1002/ece3.7191)
Supplement: Supplementary file 1 — Supplementary Material [file ECE3-11-2249-s001.docx]

**SUPPORTING INFORMATION**

**Reliability of animal counts and implications on the interpretation of trends**

David Vallecillo, Michel Gauthier-Clerc, Matthieu Guillemain, Marion Vittecoq, Philippe Vandewalle, Benjamin Roche, Jocelyn Champagnon.

**Supporting 1: Percentage of simulations that detect a decline**

| **Scenarios**  **Trends** | O1 T1  (N=24) | | | O1 T5  (N=100) | | O1 T25  (N=100) | | O24 T1  (N=100) | | O24 T5  (N=100) | | O24 T25  (N=100) | |
| --- | --- | --- | --- | --- | --- | --- | --- | --- | --- | --- | --- | --- | --- |
|  | *Tmin* | | 25 years | *Tmin* | 25 years | *Tmin* | 25 years | *Tmin* | 25 years | *Tmin* | 25 years | *Tmin* | 25 years |
| - 7.4 % per year | 100 | | 100 | 100 | 100 | 100 | 100 | 100 | 100 | 100 | 100 | 100 | 100 |
| - 3.9 % per year | 100 | 100 | | 100 | 100 | 100 | 100 | 100 | 100 | 100 | 100 | 100 | 100 |
| - 1.1 % per year | 100 | 100 | | 78.6 | 78.6 | 97 | 97 | 100 | 100 | 100 | 100 | 100 | 100 |

*Supporting Table A1. Percentage of simulations that detect a decline for Tmin and for 25 years of monitoring.*

**Supporting 2: Percentage precision**

Method: We measured the percentage precision (PP) on the confidence interval (CI) of the trend estimate as a function of the number of monitoring years compared to 3 years of monitoring.

PP $=100-\frac{\left( CI upper bound Ti-CI lower bound Ti \right)*100}{\left( CI upper bound T3-CI lower bound T3 \right)}$

where *CI upper bound Ti* corresponds to the value of the upper bound of the confidence interval of the trend estimate for all integers i between 4 and 25. *CI lower bound* corresponds to the value of the lower bound of the confidence interval of the trend estimate. *CI T_3_* corresponds to the confidence interval of the trend estimate for three monitoring years. 3 years of monitoring are the minimum required to calculate a confidence interval. For each of the 100 simulations (random noise) for each random selection of observers under the different scenarios, we evaluated the percentage precision for each monitoring duration, T_+1_, T_+2_, ..., T_+22_, compared to three years of monitoring, T_=3_.

Result: The PP did not differ within the same scenario regardless of the strength of the trend tested. For example, for the scenario O1 T1, 5 years of monitoring generated more than 80% of PP (relative to three years of monitoring) for all trends (-7.4%, -3.9% or -1.1% per year). Temporal changes in observers may lead to a loss of precision of the CI (decrease in PP values). For example, for the trend - 7.4% per year for 5 years of monitoring, scenario O1 T1 generated average PP values between 82.1 % and 88.3 % (Supporting Figure A1). In contrast, under scenarios O1 T5 and O1 T25, average PP values were lower: between 38 .7 % and 92.2 % (the average *Tmin* = 7 years) and between 76.1 % and 99.1 % (the average *Tmin* = 10 years), respectively (Supporting Figure A1).

When observers identity changed spatially (scenarios O24), the temporal changes of observers did not influence the PP for all trends tested. For example, for the trend - 7.4% per year, there was no change in PP between scenarios with spatial changes (Supporting Fig. A2). In addition, the PP values with spatial changes of observers were similar to those of scenario O1 T1 (Supporting Fig. A2). For example, after 5 years of monitoring, the average *Tmin* required to detect the trend for scenarios O1 T1, O24 T1, O24 T5, O24 T25, generated the same average PP of 85.8% ± 0.8 (s.d.).


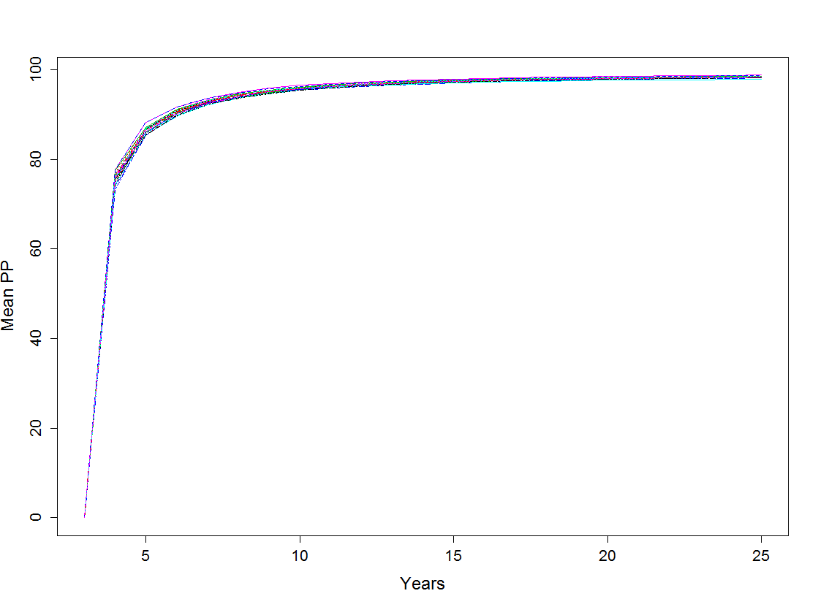


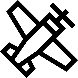

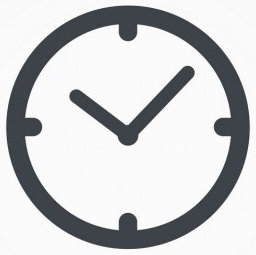


O1 T1


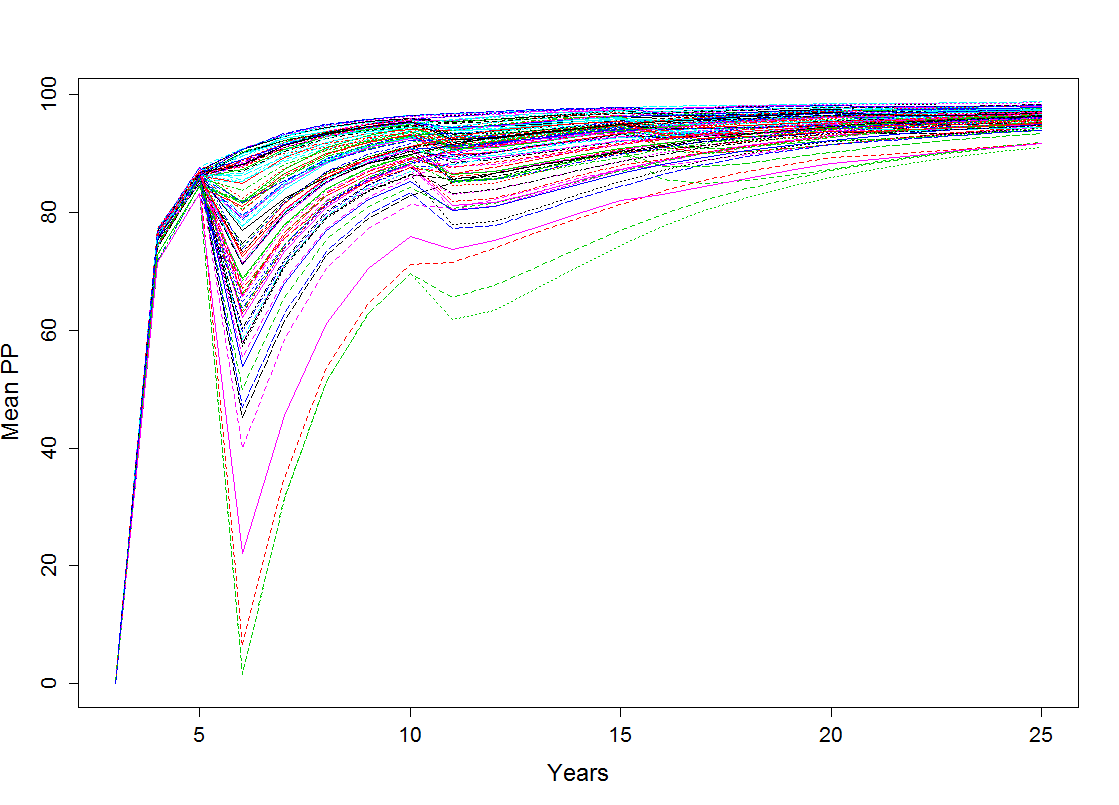


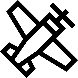

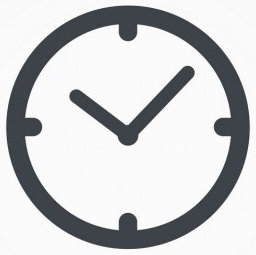

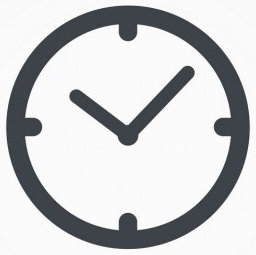


O1 T5


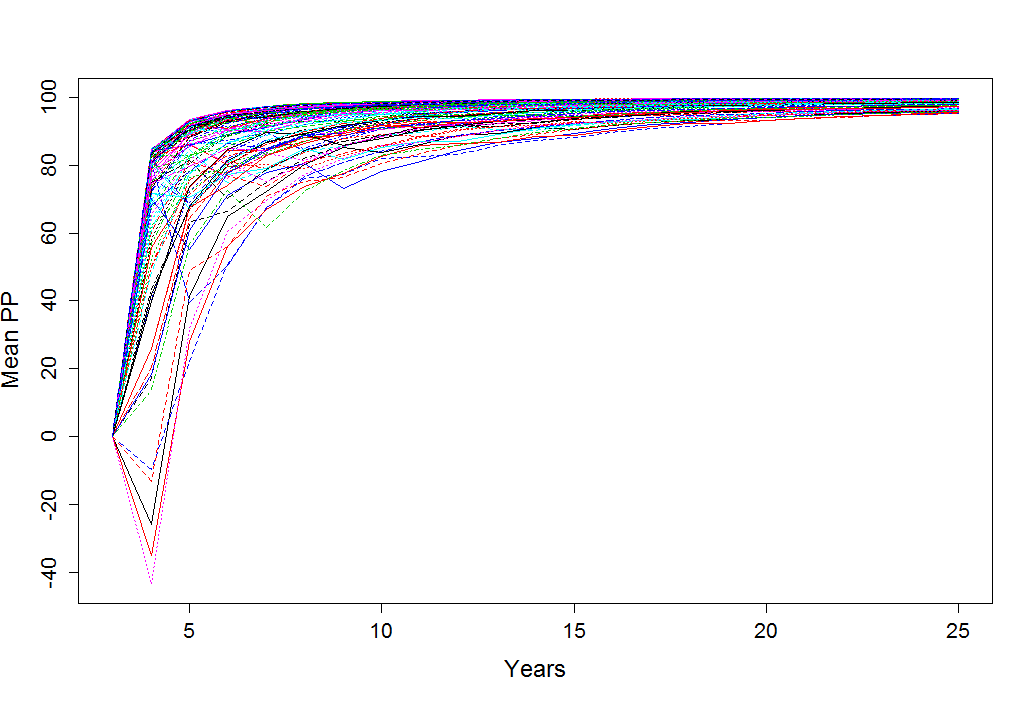


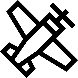

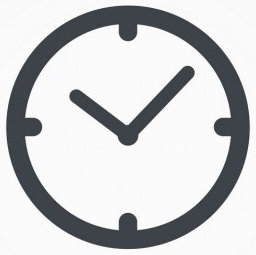

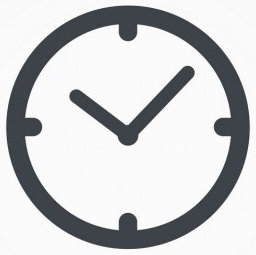

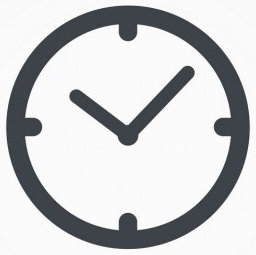


O1 T25

*Supporting Figure A1. Mean PP according to the number of years of monitoring for the trend - 7.4% per year and for all scenarios with only temporal changes in observers (100 simulations with random noise for each of the 24 observers for the scenario O1 T1 and for each of the 100 random selections of observers for scenarios O1 T5 and O1 T25).*


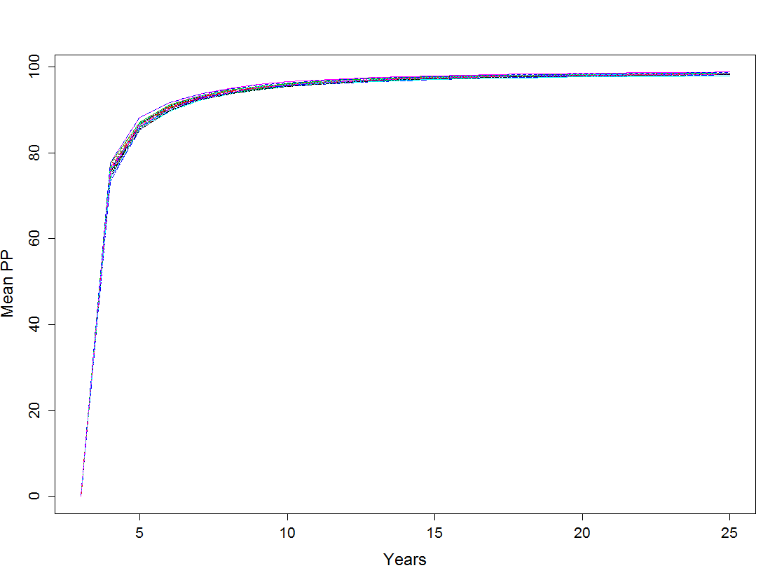


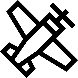

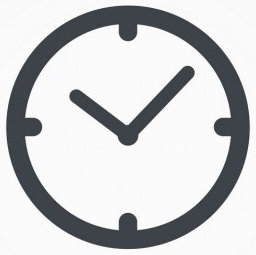


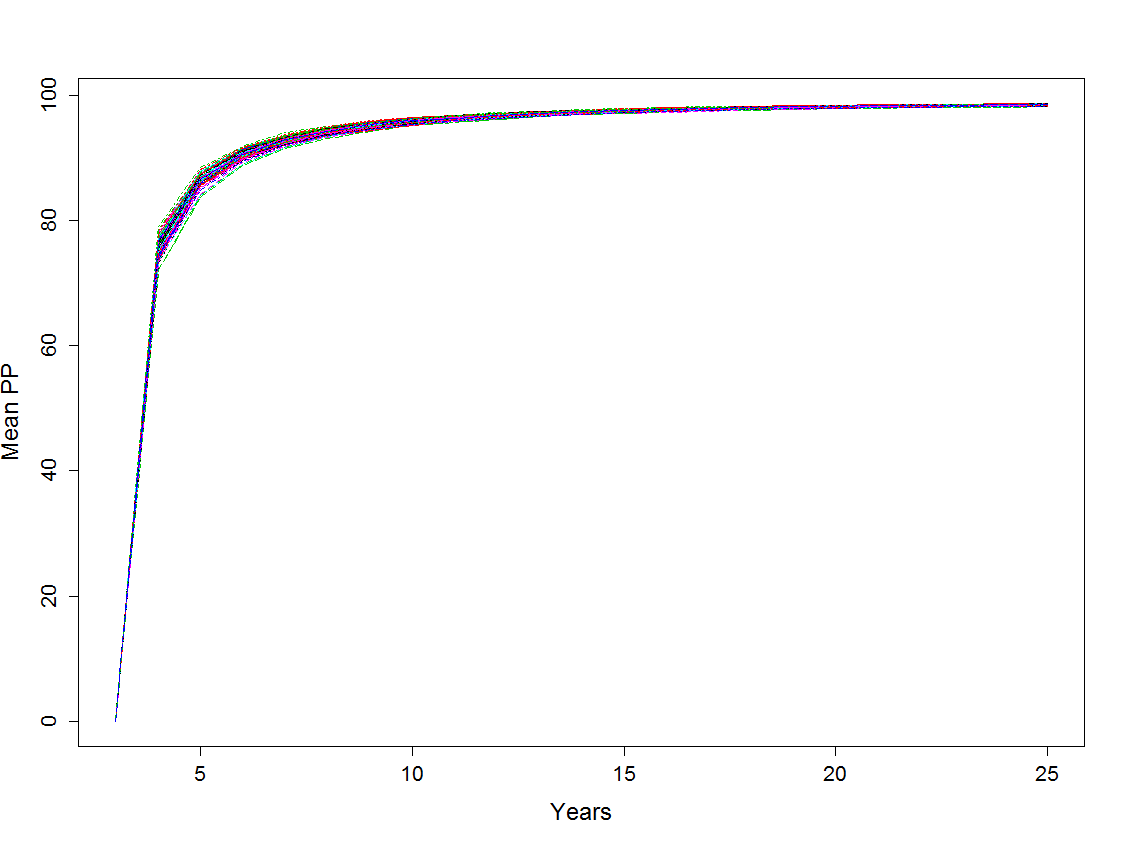


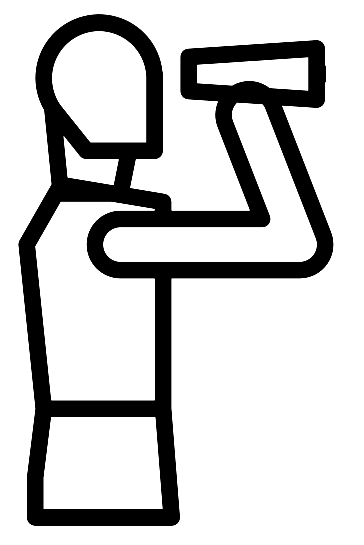

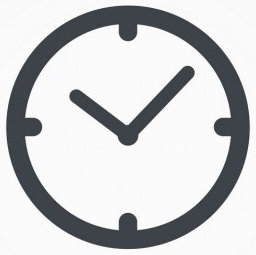


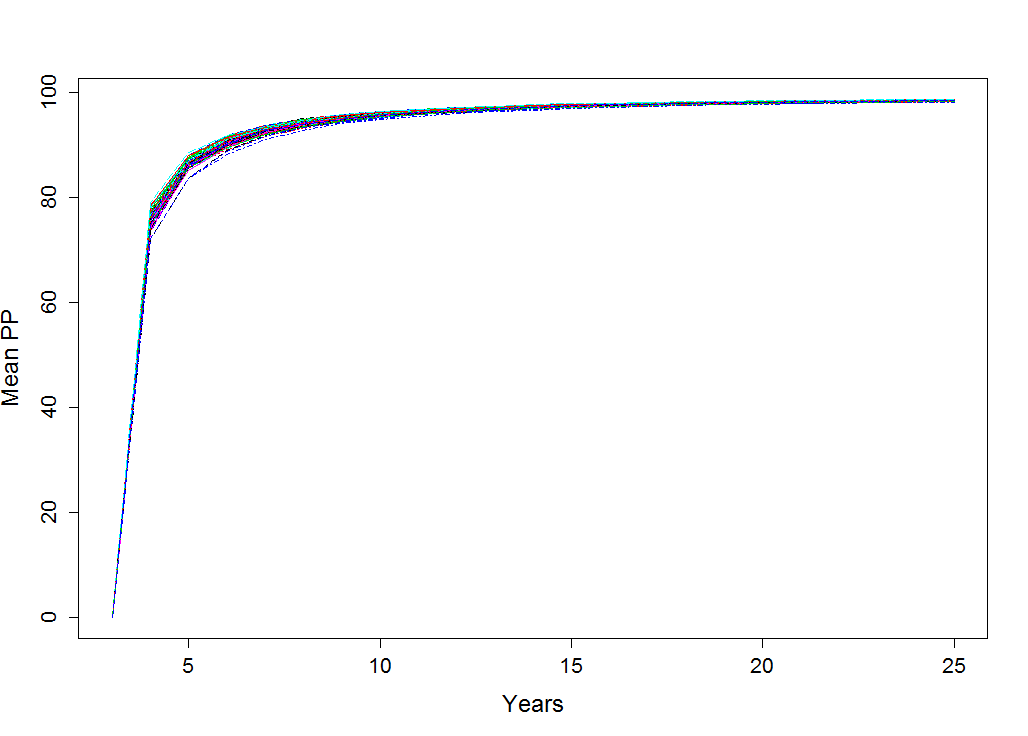


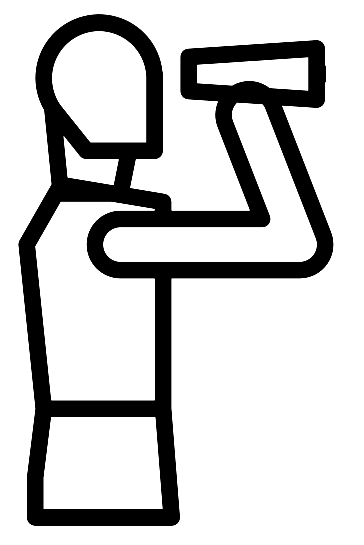

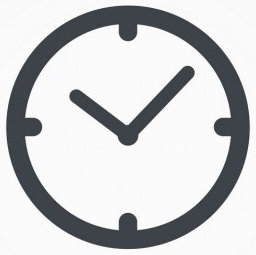

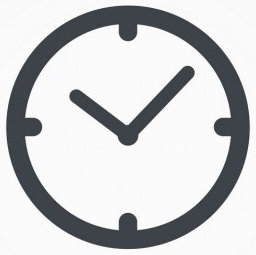


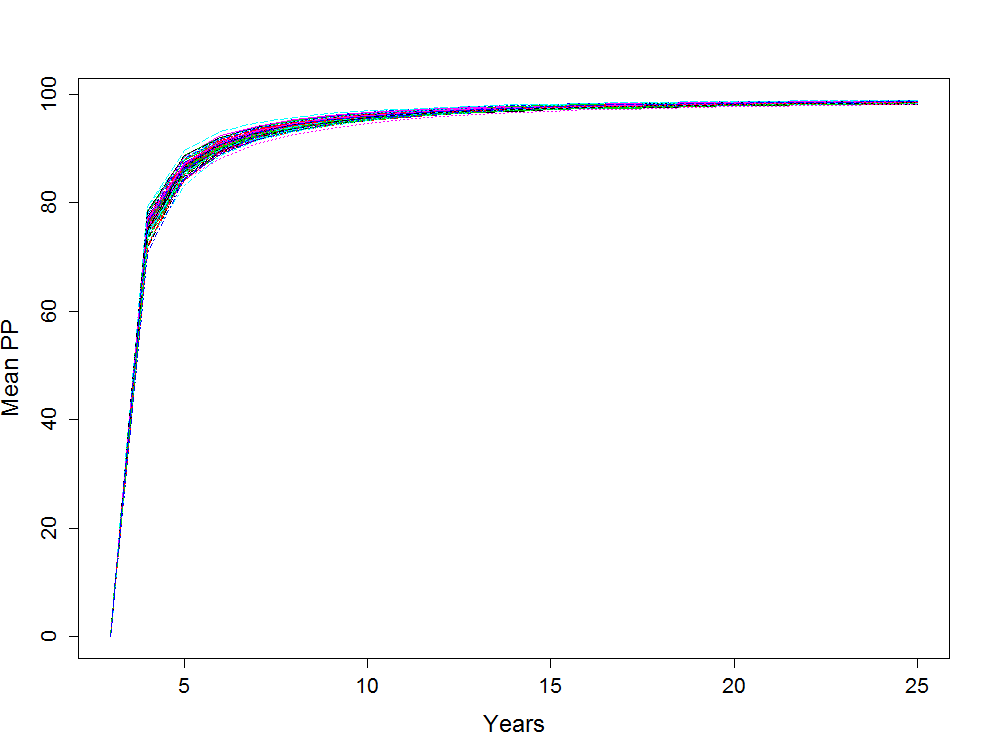


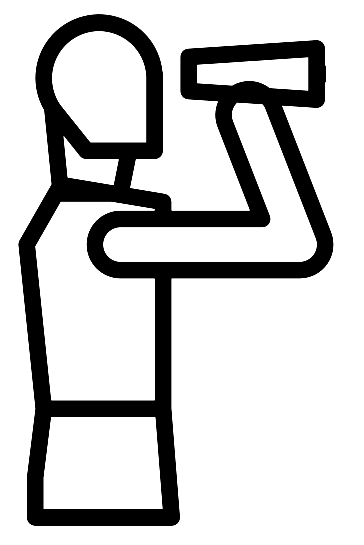

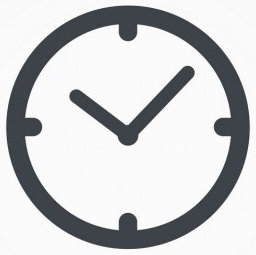

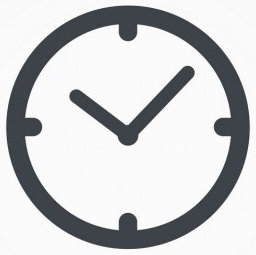

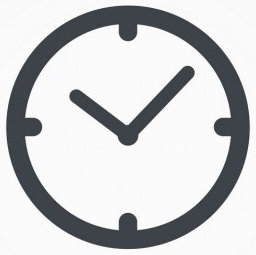


*Supporting Figure A2. Mean PP according to the number of years of monitoring for the trend - 7.4% per year and for all scenarios with spatial and temporal changes in observers (100 simulations with random noise for each of the 24 observers for the scenario O1 T1 and for each of the 100 random selections of observers for scenarios O24 T1, O24 T5 and O24 T25).*

**Supporting 3: NRMSD**


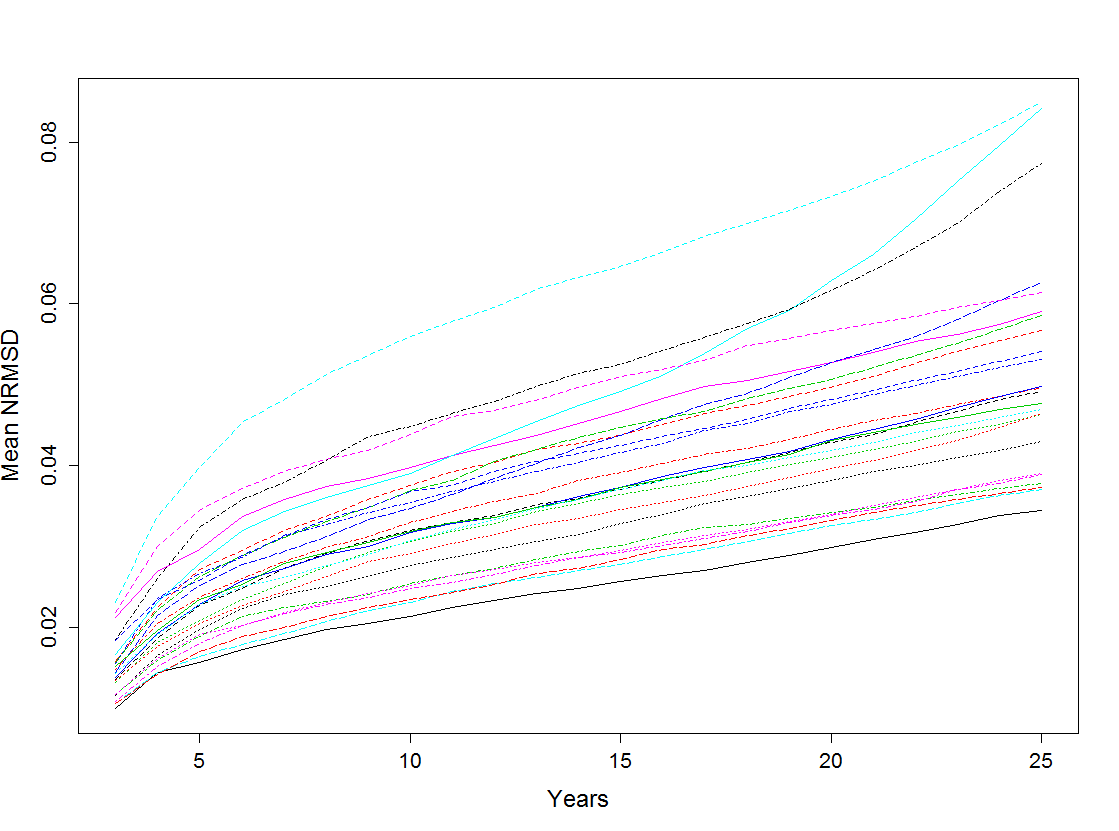


- 7.4% per year


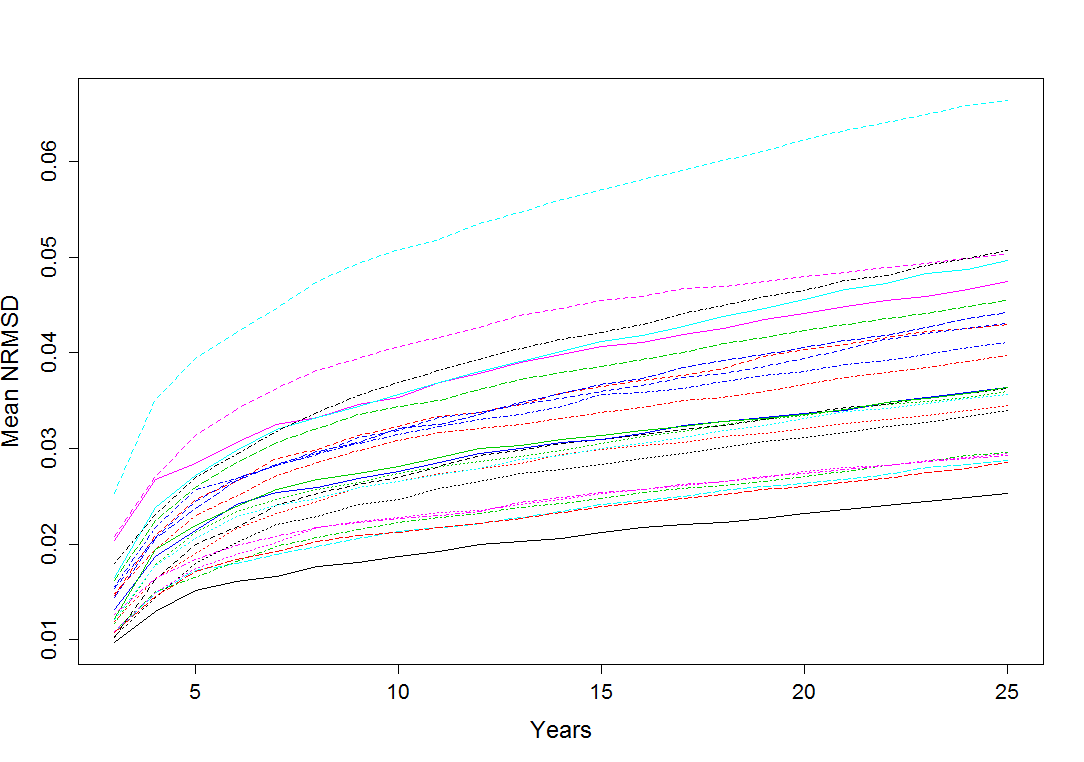


- 3.9% per year


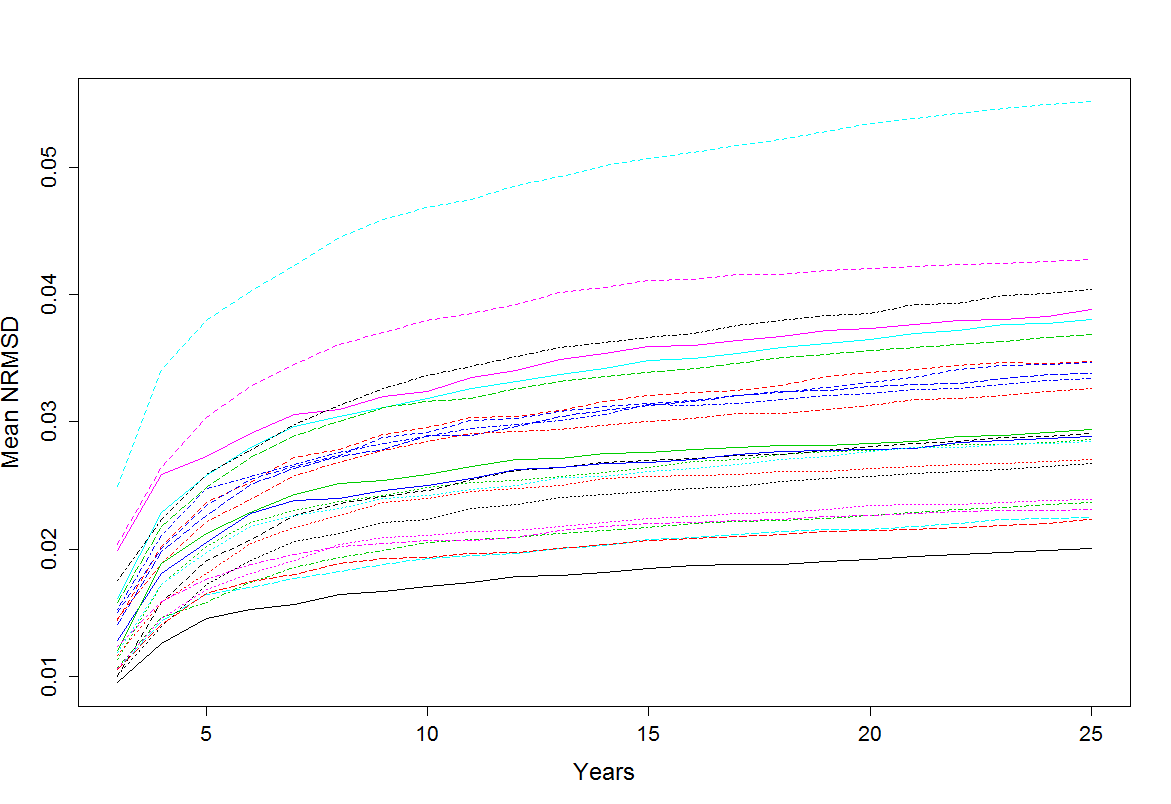


- 1.1% per year

*Supporting Figure A3. Mean NRMSD according to the number of years of monitoring for the O1 T1 scenario and for all trends tested (100 simulations with random noise for each of the 24 experienced observers).*

In this configuration (20 observers randomly counted 4 sites and 4 additional observers randomly counted 5 sites), observer spatial changes reduced the influence of observer temporal changes on NRMSD mean values. However, NRMSD mean values were lower in O1 T1 than in scenarios where observers changed spatially (Supporting Fig. A4), depending on the capacity of estimating groups among observers. In some cases, less residual variance (greater precision of the slope parameter estimate) was recorded when the observer counted all sites and over the entire monitoring period.


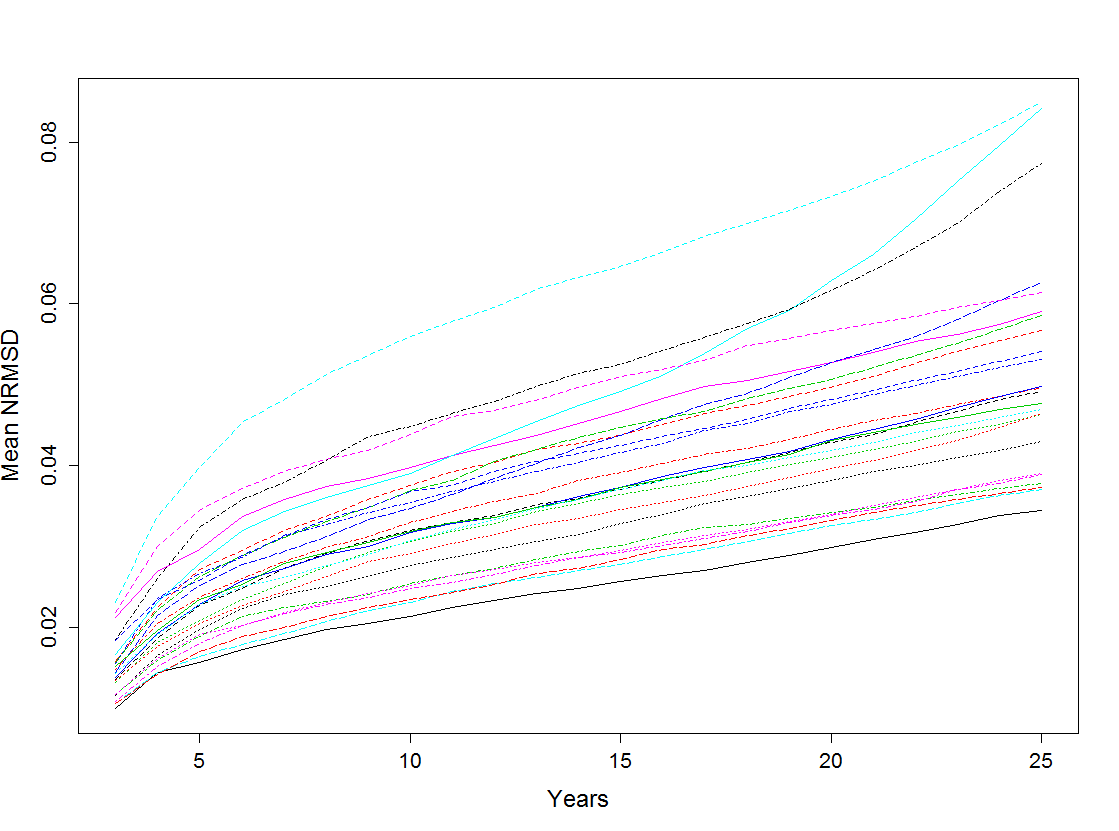


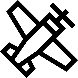

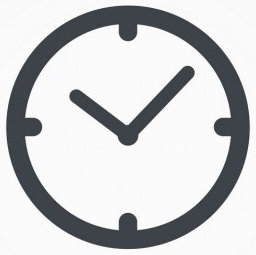


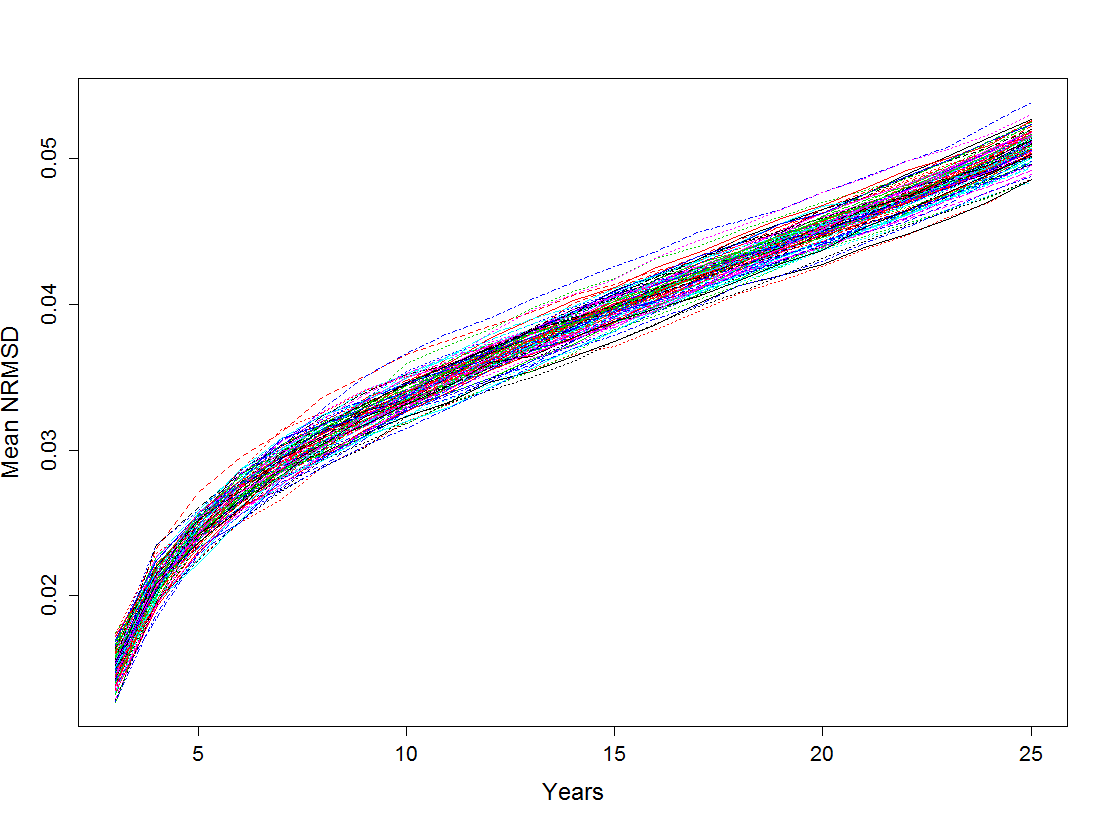


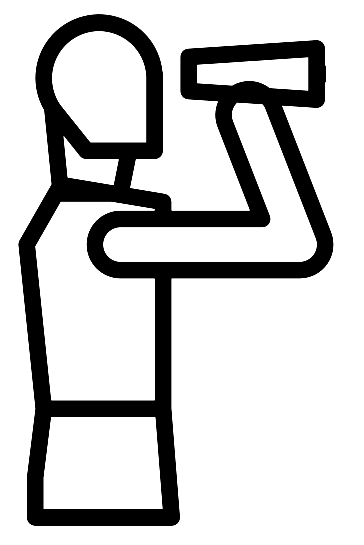

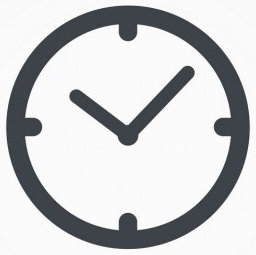


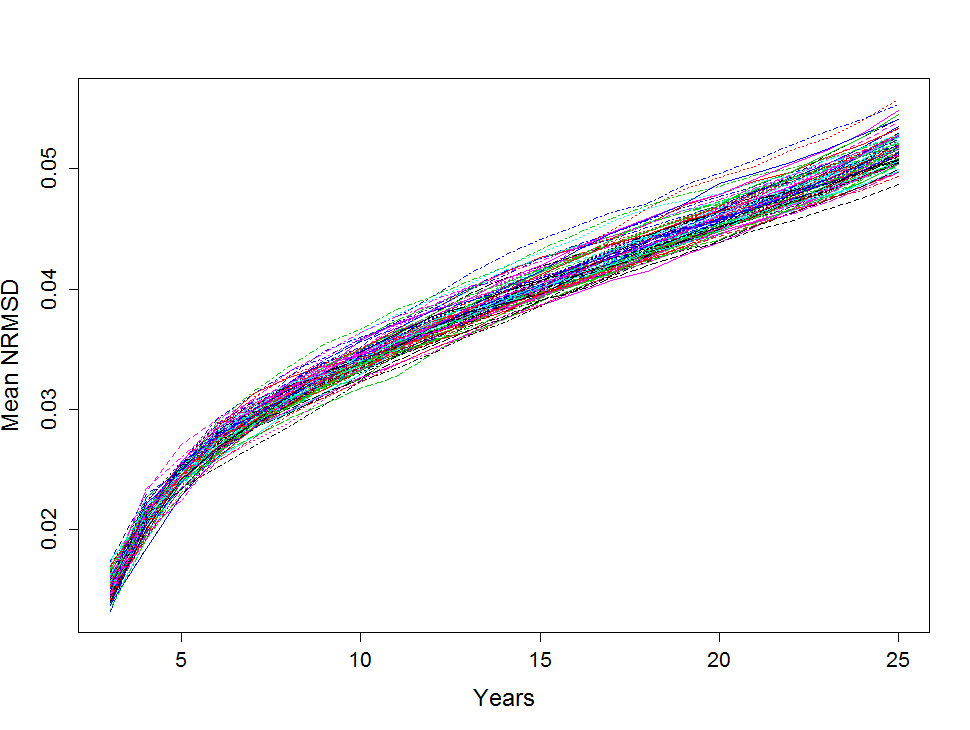


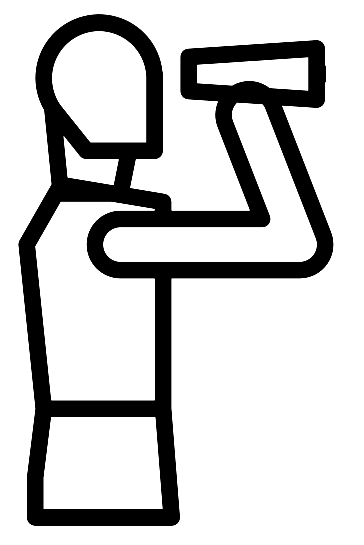

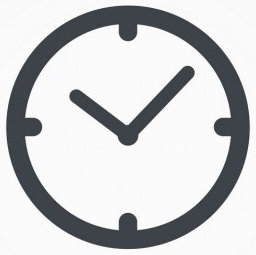

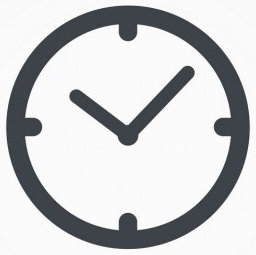


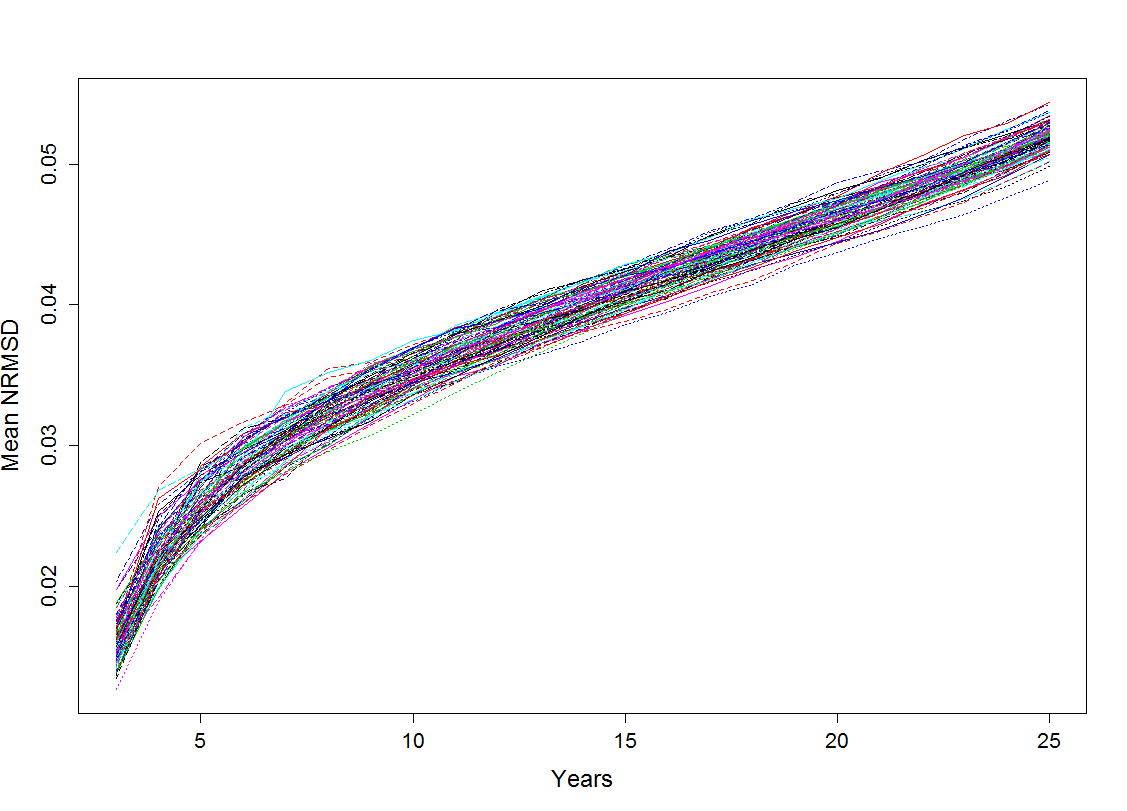


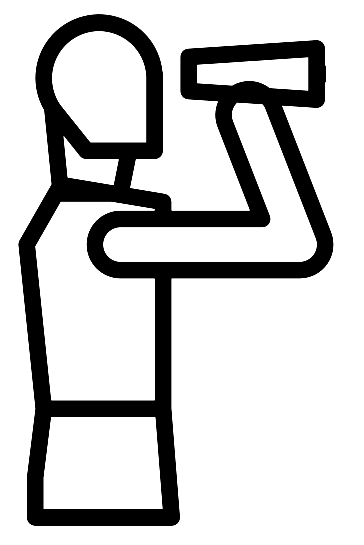

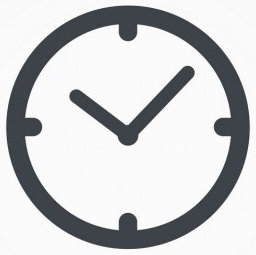

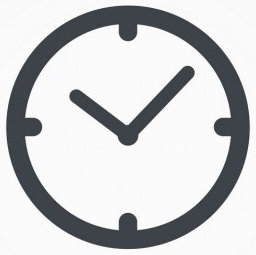

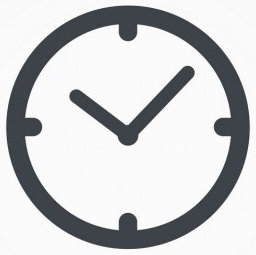


*Supporting Figure A4. Mean NRMSD according to the number of years of monitoring for the trend - 7.4% per year and for all scenarios with spatial and temporal changes in observers (100 simulations with random noise for each of the 24 observers for the scenario O1 T1 and for each of the 100 random selections of observers for scenarios O24 T1, O24 T5 and O24 T25).*

**Supporting 4: Scenario O2 T1 and Scenario O24 T1** -

NRMSD for scenarios with 2 observers spread over 100 sites (Supporting Fig. A5) and 24 observers spread over 24 sites (Supporting Fig. A6).

Scenario O2 T1


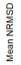

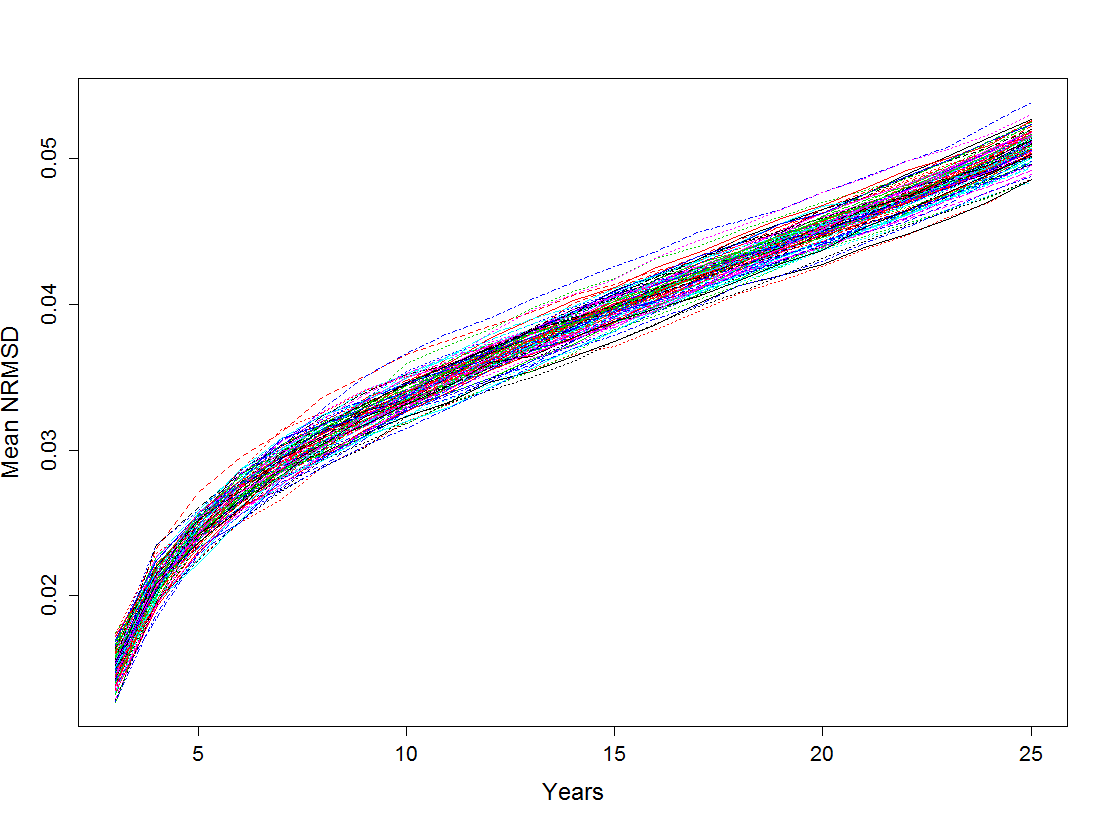


Scenario O24 T1

*Supporting Figure A5. Mean NRMSD according to the number of years of monitoring for the trend - 7.4 % per year and for scenarios with spatial changes in observers (100 simulations with random noise for each of the 100 random selections of observers for the 2 scenarios). O24 T1: 20 observers randomly counted 4 sites and 4 additional observers randomly counted 5 sites. O2 T1: 2 observers randomly counted 50 sites each.*


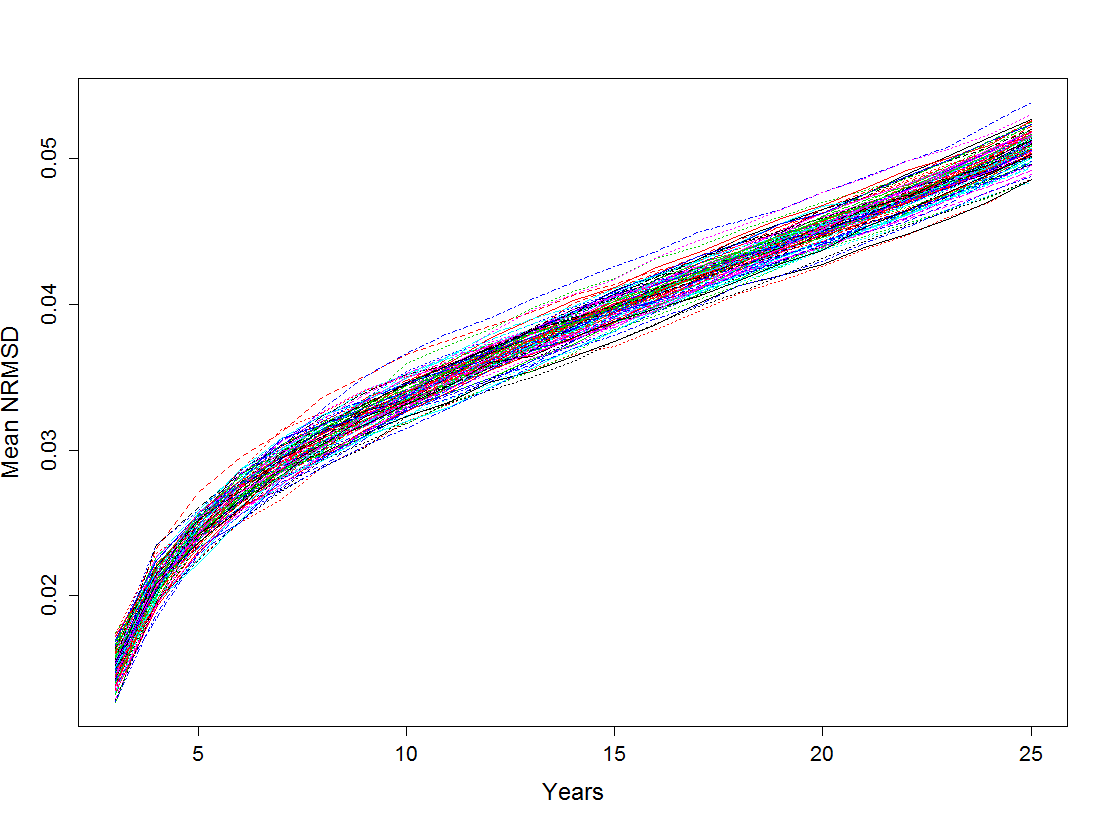


Scenario O24 T1


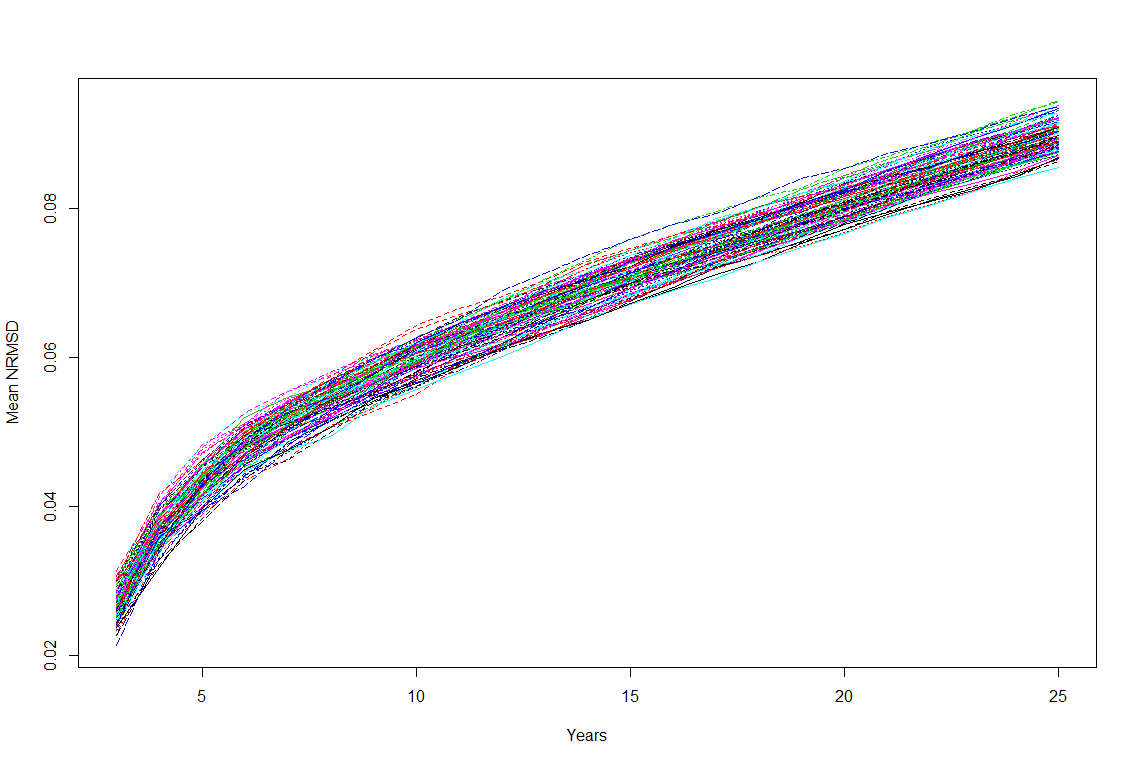


Scenario O24 T1 -

*Supporting Figure A6. Mean NRMSD according to the number of years of monitoring for the trend - 7.4 % per year and for scenarios with spatial changes in observers (100 simulations with random noise for each of the 100 random selections of observers for the 2 scenarios). O24 T1: 20 observers randomly counted 4 sites and 4 additional observers randomly counted 5 sites. O24 T1 -: 24 observers randomly counted 1 site each.*
